# Quantitative detection and reduction of potentially pathogenic bacterial groups of *Aeromonas*, *Arcobacter*, *Klebsiella pneumoniae* species complex, and *Mycobacterium* in wastewater treatment facilities

**DOI:** 10.1371/journal.pone.0291742

**Published:** 2023-09-28

**Authors:** Masataka Aoki, Yasuyuki Takemura, Shuji Kawakami, Wilasinee Yoochatchaval, Thao Tran P., Noriko Tomioka, Yoshitaka Ebie, Kazuaki Syutsubo

**Affiliations:** 1 Regional Environment Conservation Division, National Institute for Environmental Studies, Tsukuba, Ibaraki, Japan; 2 Department of Civil Engineering, National Institute of Technology (KOSEN), Nagaoka College, Nagaoka, Niigata, Japan; 3 Department of Environmental Engineering, Faculty of Engineering, Kasetsart University, Bangkok, Thailand; 4 Material Cycles Division, National Institute for Environmental Studies, Tsukuba, Ibaraki, Japan; 5 Research Center of Water Environment Technology, School of Engineering, The University of Tokyo, Bunkyo-ku, Tokyo, Japan; Purdue University, UNITED STATES

## Abstract

Water quality parameters influence the abundance of pathogenic bacteria. The genera *Aeromonas*, *Arcobacter*, *Klebsiella*, and *Mycobacterium* are among the representative pathogenic bacteria identified in wastewater. However, information on the correlations between water quality and the abundance of these bacteria, as well as their reduction rate in existing wastewater treatment facilities (WTFs), is lacking. Hence, this study aimed to determine the abundance and reduction rates of these bacterial groups in WTFs. Sixty-eight samples (34 influent and 34 non-disinfected, treated, effluent samples) were collected from nine WTFs in Japan and Thailand. 16S rRNA gene amplicon sequencing analysis revealed the presence of *Aeromonas*, *Arcobacter*, and *Mycobacterium* in all influent wastewater and treated effluent samples. Quantitative real-time polymerase chain reaction (qPCR) was used to quantify the abundance of *Aeromonas*, *Arcobacter*, *Klebsiella pneumoniae* species complex (KpSC), and *Mycobacterium*. The geometric mean abundances of *Aeromonas*, *Arcobacter*, KpSC, and *Mycobacterium* in the influent wastewater were 1.2 × 10^4^–2.4 × 10^5^, 1.0 × 10^5^–4.5 × 10^6^, 3.6 × 10^2^–4.3 × 10^4^, and 6.9 × 10^3^–5.5 × 10^4^ cells mL^−1^, respectively, and their average log reduction values were 0.77–2.57, 1.00–3.06, 1.35–3.11, and −0.67–1.57, respectively. Spearman’s rank correlation coefficients indicated significant positive or negative correlations between the abundances of the potentially pathogenic bacterial groups and *Escherichia coli* as well as water quality parameters, namely, chemical/biochemical oxygen demand, total nitrogen, nitrate-nitrogen, nitrite-nitrogen, ammonium-nitrogen, suspended solids, volatile suspended solids, and oxidation-reduction potential. This study provides valuable information on the development and appropriate management of WTFs to produce safe, hygienic water.

## Introduction

Wastewater treatment facilities (WTFs) are vital components in reducing pathogens in untreated wastewater [1]. The use of reclaimed water derived from treated wastewater for agricultural irrigation; environmental enhancement; groundwater recharge; portable reuse; and industrial, recreational, and urban use is gaining attention owing to rapid urbanization, especially in developing countries, and the increasing global water scarcity [2]. Jones et al. estimated that 40.7 × 10^9^ m^3^ year^−1^ of treated wastewater is intentionally reused for human purposes [3]. A major concern regarding the discharge of wastewater into the environment and the use of treated wastewater is the inadequate removal of pathogenic microorganisms. Active pathogenic microorganisms in biologically treated wastewater can be effectively removed using ultraviolet light, ozonation, or chlorination disinfection [4]. Although chlorination disinfection is the most common and cost-effective option, it produces mutagenic/carcinogenic disinfection by-products, such as trihalomethanes and haloacetic acids [5]. As high pathogen reduction rates lead to low final disinfectant demand [6], the development and appropriate management of WTFs with high pathogen reduction rates are crucial to reduce not only disinfection costs but also the formation of disinfection by-products.

Recent advances in high-throughput DNA sequencing approaches (e.g., 16S ribosomal RNA [rRNA] gene amplicon sequencing and metagenomic sequencing) have enabled researchers to reveal the phylogenetic diversity, relative abundance, and putative pathogenic traits of potentially pathogenic bacteria in wastewater environments [7–10]. Representative and frequently observed potentially pathogenic bacteria in wastewater, identified by conventional cultivation and high-throughput DNA sequencing approaches, include those from the genera *Aeromonas*, *Arcobacter*, *Klebsiella*, and *Mycobacterium* [7–11]. Galagoda et al. recently investigated the dynamics of potentially pathogenic bacteria in a Japanese municipal WTF using quantitative 16S rRNA gene sequencing and a novel pathogen database [10]. While 69 potentially pathogenic bacterial genera were detected in the influent wastewater, only 13 potentially pathogenic bacterial genera, including abundant levels of *Aeromonas*, *Arcobacter*, *Klebsiella*, and *Mycobacterium*, were quantitatively detected in the chlorine-disinfected, treated effluent. These data indicate the potential importance of better understanding the abundance and persistence of these potentially pathogenic bacteria in WTFs to reduce health risks associated with treated wastewater. However, to the best of our knowledge, the group-level abundance and persistence of *Aeromonas*, *Arcobacter*, *Klebsiella*, and *Mycobacterium* in the existing WTFs in East Asian regions remain largely unknown owing to limited investigation [10, 12].

The genus *Aeromonas* belongs to the family *Aeromonadaceae* within the phylum *Pseudomonadota* (formerly *Proteobacteria*) and inhabits various aquatic environments [13]. *Aeromonas* species are important human pathogens capable of causing various diseases, including gastroenteritis as well as blood-borne, skin, soft tissue, intra-abdominal, respiratory tract, urogenital tract, and eye infections [13, 14]. The most common human pathogenic *Aeromonas* species are *Ae*. *hydrophila*, *Ae*. *caviae*, *Ae*. *veronii*, and *Ae*. *dhakensis* [15, 16]. Biological wastewater treatment can reduce the abundance of *Aeromonas*. For instance, a 1.14 and 2.57 log reduction of *Aeromonas* was reported in a Moroccan basin wastewater treatment system [17] and a Brazilian sanitary sewage stabilization pond [18], respectively, after biological treatment.

Members of the genus *Arcobacter* are aerotolerant *Campylobacter*-like bacteria with a psychrotrophic nature and currently belong to the family *Arcobacteraceae* within the phylum *Campylobacterota* (formerly *Epsilonproteobacteria*). They are zoonotic food and waterborne pathogens that cause gastroenteritis and bacteremia in humans [19]. The majority of *Arcobacter* infections in humans and animals are caused by *Ar*. *butzleri*, *Ar*. *cryaerophilus*, *Ar*. *skirrowii*, *and Ar*. *thereius* [19]. Virulence genes responsible for adhesion, invasion, and cytotoxicity have been reported not only in the *Arcobacter* species mentioned above but also in other taxonomically diverse *Arcobacter* members [20, 21]. Although the predominant mode of transmission is contaminated food, contaminated water is also a potential source of *Arcobacter* infection [21]. In the existing WTFs in Beijing, Denmark, and Southern Arizona, *Arcobacter* reductions ranging from 1 to 3 logs were confirmed through molecular biological analyses [9, 12, 22].

The genus *Klebsiella* belongs to the family *Enterobacteriaceae* within the phylum *Pseudomonadota*. Environmental *Klebsiella* isolates have pathogenic potential [23, 24], and natural aquatic environments are potential reservoirs for the growth and spread of *Klebsiella* species [25]. The *K*. *pneumoniae* species complex (KpSC) generally refers to closely related *Klebsiella* species that share a 95–96% average nucleotide identity with *K*. *pneumoniae*, a causative agent of pneumonia, urinary tract infections, and wound infections [26]. KpSC includes seven phylogroups: *K*. *pneumoniae* (Kp 1), *K*. *quasipneumoniae* subsp. *quasipneumoniae* (Kp 2), *K*. *variicola* subsp. *variicola* (Kp 3), *K*. *quasipneumoniae* subsp. *similipneumoniae* (Kp 4), *K*. *variicola* subsp. *tropica* (Kp 5), *K*. *quasivariicola* (Kp 6), and *K*. *africana* (Kp 7). All KpSC bacteria are potentially pathogenic to humans and animals, and some strains associated with this group are hypervirulent and/or antibiotic-resistant strains [27]. In a municipal WTF, a 4.23–4.33 log reduction of *K*. *pneumoniae* was confirmed through a *K*. *pneumoniae-*specific quantitative real-time polymerase chain reaction (qPCR) assay [28]. However, to the best of our knowledge, the group-level abundance and persistence of KpSC in the existing WTFs have not been reported yet owing to the general lack of their quantification before the development of a highly specific qPCR assay, as reported by Barbier et al. [27].

The genus *Mycobacterium* belongs to the family *Mycobacteriaceae* within the phylum *Actinomycetota* (formerly *Actinobacteria*). *Mycobacterium* species are generally classified into two distinct groups: the *Mycobacterium tuberculosis* complex (MTC) and nontuberculous mycobacteria (NTM) [29]. In humans, MTC members cause pulmonary/extrapulmonary tuberculosis, whereas NTM members cause major clinical diseases, such as chronic pulmonary disease, disseminated disease in severely immunocompromised patients, skin and soft tissue infections, and superficial lymphadenitis, particularly cervical lymphadenitis in children [30]. NTM are known as “environmental mycobacteria” owing to their ubiquitous distribution in the environment [29, 30]. The rate of NTM infections is increasing, with the possibility of turning serious in the most vulnerable individuals [31]. Radomski et al. developed a specific qPCR assay for the genus *Mycobacterium* [32]. This qPCR assay was later applied to reveal the behavior of *Mycobacterium* in a WTF located in Paris, which used a combined process of physical-chemical decantation followed by biofiltration [33]. The reported data showed that most of the *Mycobacterium* population in the WTF influent was removed by a physical–chemical decantation process (2.4 log reduction) and then completely removed through subsequent biofiltration.

Indicators for fecal contamination, such as *Escherichia coli* and total fecal coliforms, have been used as water quality criteria for the use of reclaimed or recycled water and to adhere to environmental and effluent standards [34–37]. Many previous studies have used these indicators to assess the pathogen reduction performance of WTFs [1]. However, poor positive correlations between the presence/abundance of fecal indicators and pathogenic bacteria have been reported in several water environments [17, 38–41]. In addition, water quality parameters can influence the abundance of pathogenic bacteria in water environments [17, 33, 40]. For instance, Boussaid et al. revealed a significant negative correlation between *Aeromonas* abundance and chemical oxygen demand (COD) in the treated effluent of a Moroccan basin wastewater treatment system [17]. Furthermore, *Mycobacterium* abundance in a WTF in Paris was related to the COD, biochemical oxygen demand (BOD), orthophosphates, and suspended solids (SS) concentrations [33]. Information on such correlations could be useful to develop and appropriately manage WTFs with high pathogen reduction rates; however, such correlation data in wastewater and related environments are limited. Therefore, this study aimed to determine the abundance and reduction rates of the above-mentioned four potentially pathogenic bacterial groups in the influent wastewater and treated effluent of existing WTFs in Japan and Thailand. In addition, we aimed to reveal the correlation among the abundances of *E*. *coli*, the potentially pathogenic bacterial groups, and various water quality parameters.

## Materials and methods

### Collection of wastewater samples

Wastewater sampling from six decentralized WTFs (A, B, C, D, E, and F) in Tokushima, Japan, was conducted between November 2021 and August 2022. Wastewater sampling from three centralized domestic WTFs (G, H, and I) in Bangkok, Thailand, was conducted between November 2020 and December 2021. The nine investigated WTFs are summarized in Table 1. WTFs A and B were small-scale on-site domestic WTFs, known as the *Johkasou* system [42], that treated domestic wastewater from individual, private houses (design wastewater flow: 1.0 and 1.4 m^3^ day^−1^, respectively). WTF C was a rural community WTF that used a combined system of an anaerobic filter bed and contact aeration (design wastewater flow: 164.7 m^3^ day^−1^). WTFs D, E, and F were small-scale WTFs, known as community plants, that treated domestic wastewater from different housing complexes (design wastewater flow: 266, 400, and 850 m^3^ day^−1^, respectively). WTF D used a contact aeration system, whereas WTFs E and F used an extended aeration system. WTFs G, H, and I were centralized domestic WTFs that used contact stabilization activated sludge, cyclic activated sludge, and activated sludge with nutrient removal systems, respectively [43]. The treatment capacities of WTFs G, H, and I were 30,000, 200,000, and 350,000 m^3^ day^−1^, respectively. Tertiary treatment based on chemical precipitation was not applied in any of the analyzed WTFs. A total of 68 grab samples were collected, consisting of 34 influent wastewater and 34 treated effluent samples. The WTFs located in Tokushima, Japan, considered for this study routinely used chlorination to disinfect treated wastewater before discharging to the environment; however, we collected the treated effluent samples before the disinfection process. The treated effluents from WTFs G, H, and I in Bangkok, Thailand, were not subjected to any disinfection process [44]. Wastewater sampling was permitted by the owners of the *Johkasou* systems; the Environment Division, Naka-cho, Tokushima Prefecture; the Environmental Preservation Division, Tokushima City, Tokushima Prefecture; and the Drainage and Sewage Department, Bangkok Metropolitan Administration, Bangkok.

**Table 1:** Overview of the wastewater treatment facilities included in the study.

Facility	*Johkasou* A	*Johkasou* B	Rural community wastewater treatment facility C
System	Impurity removal and a fluidized bed biofilter	Anaerobic filter beds and a fluidized bed biofilter	Combined system of an anaerobic filter bed and contact aeration
Location	Tokushima, Japan	Tokushima, Japan	Tokushima, Japan
Design wastewater flow/ treatment capacity (m^3^ day^−1^)	1.0	1.4	164.7
Sewered population	4 ^a^	5 ^a^	610
Sampling time	Nov 2021; Feb, May, and Aug 2022	Nov 2021; Feb, May, and Aug 2022	Nov 2021; Feb, May, and Aug 2022
Facility	Community plant D	Community plant E	Community plant F
System	Contact aeration	Extended aeration	Extended aeration
Location	Tokushima, Japan	Tokushima, Japan	Tokushima, Japan
Design wastewater flow/ treatment capacity (m^3^ day^−1^)	266	400	850
Sewered population	688	1,000	5,000
Sampling time	Nov 2021; Feb, May, and Aug 2022	Nov 2021; Feb, May, and Aug 2022	Nov 2021; Feb, May, and Aug 2022
Facility	Centralized domestic wastewater treatment facility G	Centralized domestic wastewater treatment facility H	Centralized domestic wastewater treatment facility I
System	Contact stabilization activated sludge	Cyclic activated sludge	Activated sludge with nutrient removal
Location	Bangkok, Thailand	Bangkok, Thailand	Bangkok, Thailand
Design wastewater flow/ treatment capacity (m^3^ day^−1^)	30,000	200,000	350,000
Sewered population	120,000	580,000	1,080,000
Sampling time	Nov 2020; Sep, Oct, and Dec 2021	Nov 2020; Sep and Oct 2021	Nov 2020; Sep and Oct 2021

^a^ The number of people living in the house where the *Johkasou* system was installed.

### Wastewater sample filtration and genomic DNA extraction

Filtration of wastewater samples and subsequent genomic DNA extraction were performed as described previously [40]. Briefly, wastewater subsamples (40 mL each) were initially fixed by adding 10 mL of ethanol. Next, 2.5–10 mL of ethanol-fixed samples (net sample filtration volume: 2.0–8.0 mL) were filtered through an Omnipore polytetrafluoroethylene membrane filter (diameter, 25 mm; pore size, 0.2 μm; Merck KGaA, Darmstadt, Germany), and the filters were stored at −80 °C until DNA extraction. DNA extraction from the resulting filters was performed using an Extrap Soil DNA Kit Plus ver.2 (BioDynamics Laboratory Inc., Tokyo, Japan) according to the manufacturer’s instructions. As a slight modification, the final DNA elution volume in TE buffer (10 mM Tris-HCl and 1 mM EDTA [pH 8.0]) was set to 50 μL. The concentrations of the extracted DNA were determined using the Qubit dsDNA HS Assay Kit and the Qubit 4 Fluorometer (Thermo Fisher Scientific K.K., Tokyo, Japan).

### PCR primer design for quantitative detection of *Aeromonas*

A new PCR primer set Aero581F/Aero848R, targeting the 16S rRNA gene of the genus *Aeromonas*, was designed using the probe design function of ARB v.6.0.6 [45] with the All-Species Living Tree Project (LTP) database (release 132) [46]. The *in silico* coverage and specificity of the primer set Aero581F/Aero848R and previously reported primer sets targeting the 16S rRNA gene of the genus *Aeromonas* were verified using TestPrime v.1.0 [47] against the SILVA reference database (release 138.1) (sequence collection: non-redundant [RefNR] and LTP) [46, 48].

### qPCR assays

qPCR assays were performed using the oligonucleotide primer/probe sets listed in Table 2 [27, 32, 40]. The detailed procedures of qPCR assays, qPCR data processing, and the determination of the limit of detection (LoD) and limit of quantification (LoQ) of the qPCR assays are described in S1 Text. All standard DNA fragments used in the qPCR assays were synthesized by Eurofins Genomics K.K. (Tokyo, Japan), and the sequences are listed in S1 Table. The determined LoD values for *Aeromonas*, *Arcobacter*, KpSC, and *Mycobacterium* quantification were 0.31, 0.63, 3.1, and 3.1 cells mL-sample^−1^, respectively, whereas the determined LoQ values were 3.1, 6.3, 31, and 31 cells mL-sample^−1^, respectively. Abundances below the LoQ were assigned a value of LoQ/2 for the calculation of geometric mean abundances and reduction rates of the potentially pathogenic bacterial groups.

**Table 2:** Oligonucleotide primer/probe sets for quantitative real-time polymerase chain reaction (qPCR) assays.

Target group (Target gene/region)	Primer/probe (concentration)	Name	Sequence (5ʹ to 3ʹ)	qPCR condition ^c^	PCR efficiency ^d^	Reference
Genus *Aeromonas* (16S rRNA gene)	Primer (0.20 μM)	Aero581F	GCAGGCGGTTGGATAAGTTAG	40 cycles at 95 °C for 10 s, 64 °C for 10 s, and 72 °C for 30 s	1.87–1.93	This study
		Aero848R	GTCTCAAGGACACAGCCTC			
Genus *Arcobacter* (16S rRNA gene)	Primer (0.40 μM)	Arco605F	GAAGTGAAATCCTATAGCTTAAC	40 cycles at 95 °C for 10 s, 59 °C for 10 s, and 72 °C for 15 s	1.88–1.95	[40]
		Arco688R	CGCAATCGGTATTCCTTCTGAT			
*Klebsiella pneumoniae* species complex (intergenic region of zinc uptake regulator and hemolysin genes)	Primer (0.20 μM)	ZKIR_F	CTAAAACCGCCATGTCCGATTTAA	40 cycles at 95 °C for 10 s, 60 °C for 10 s, and 72 °C for 10 s	1.83–1.89	[27]
		ZKIR_R	TTCCGAAAATGAGACACTTCAGA			
Genus *Mycobacterium* (16S rRNA gene)	Primer (0.30 μM)	110F	CCTGGGAAACTGGGTCTAAT	40 cycles at 95 °C for 10 s and 60 °C for 60 s	1.92–1.98	[32]
		I571R	CGCACGCTCACAGTTA			
	Probe (0.10 μM)	H19Rm ^a^, ^b^	TTTCACGAACAACGCGACAAAC			

^a^ The probe sequence was slightly modified from the original H19R probe sequence.

^b^ The 5ʹ and 3ʹ ends of the oligonucleotide probe were labeled with 6-carboxyfluorescein and Black Hole Quencher 1, respectively.

^c^ Each PCR program was preceded by an initial denaturation step at 95 °C for 5 min.

^d^ The PCR efficiency was calculated using the slope of the standard curve: 10^−1/slope^.

### 16S rRNA gene amplicon sequencing analysis

Amplicon sequencing was performed targeting the prokaryotic 16S rRNA gene hypervariable region V4. The analyzed influent wastewater and treated effluent samples from the six WTFs in Japan (i.e., A, B, C, D, E, and F) were collected in November 2021, whereas those from the three WTFs in Thailand (i.e., G, H, and I) were collected in October 2021. 16S rRNA gene amplicon libraries were prepared using a two-step tailed PCR method with the 515F/806R primer set [49], as described previously [50]. Briefly, the first PCR targeting the 16S rRNA genes in the genomic DNA extracts obtained using the above-mentioned DNA extraction method was performed using TaKaRa Ex *Taq* HS (Takara Bio Inc., Shiga, Japan). Thermal cycling for the first PCR consisted of an initial denaturation at 94 °C for 2 min, followed by 30 amplification cycles (94 °C for 30 s, 50 °C for 30 s, and 72 °C for 30 s) and a final extension step at 72 °C for 5 min. The first PCR products were purified using the AMPure XP reagent (Beckman Coulter Inc., Brea, CA, USA), and the purified products were subsequently subjected to a second PCR using primers with unique index sequences [50]. 16S rRNA gene amplicon sequencing was performed on a MiSeq system using a MiSeq Reagent Kit v3 (Illumina, Inc., San Diego, CA, USA) at the Bioengineering Lab. Co., Ltd., Kanagawa, Japan.

The resulting sequence data were initially subjected to primer trimming using Cutadapt v.4.1 with default parameters [51]. Sequence processing was then performed with mothur v.1.48.0 [52] using the SILVA reference database (release 138.1) [48], following MiSeq standard operating procedures [53]. The assembly and quality filtering of the paired-end amplicon reads were performed using the “make.contigs” command with the following options: maxambig = 0, maxlength = 275, maxhomop = 8, and trimoverlap = t. For operational taxonomic unit (OTU)-based analysis, the obtained sequences were clustered into OTUs at 97% sequence similarity using the OptiClust clustering algorithm [54]. The closest cultured relatives of dominant *Aeromonas*, *Arcobacter*, *Pseudarcobacter*, and *Mycobacterium* OTUs were determined using the EzBioCloud 16S-based ID service with the 16S database v.2021.07.07 [55] and the representative sequences of the dominant OTUs. The habitat preferences of the dominant OTUs were analyzed using the ProkAtlas Online Toolkit with default parameters [56].

### Water quality analysis

Water quality analysis of the influent wastewater and treated effluent samples collected from the six WTFs located in Tokushima, Japan (i.e., A, B, C, D, E, and F) was performed as described below. Water temperature, pH, and dissolved oxygen (DO) concentrations were measured using MM-42DP portable water quality meters connected to a pH probe MM4-PH or DO probe MM4-DDO (DKK-TOA CORPORATION, Tokyo, Japan). The oxidation-reduction potential (ORP) values were measured using an MM-41DP portable water quality meter connected to an ORP probe MM4-ORP (DKK-TOA CORPORATION). COD determined using the potassium dichromate method (COD_Cr_), BOD, total nitrogen (TN), ammonium-nitrogen (NH_4_^+^-N), nitrite-nitrogen (NO_2_^−^-N), nitrate-nitrogen (NO_3_^−^-N), SS, and volatile suspended solids (VSS) concentrations were measured according to the Japanese Industrial Standards (JIS) K 0102 methods [57]. The water quality parameters of the influent wastewater and treated effluent samples collected from the three WTFs in Bangkok, Thailand (i.e., G, H, and I) were determined as described previously [44, 58].

### Measurement of colony-forming units (CFUs) of *E*. *coli*

The CFUs of *E*. *coli* of all analyzed samples were determined through the Compact Dry “Nissui” *E*. *coli*/Coliform Count (EC) method (Nissui Pharmaceutical Co., Ltd., Tokyo, Japan) [59]. In brief, we observed the formation of blue or bluish-purple colored *E*. *coli* colonies in selective medium containing two chromogenic enzyme substrates: 5-bromo-4-chloro-3-indoxyl-β-D-glucuronic acid cyclohexylammonium salt (X-Gluc) and 5-bromo-6-chloro-3-indoxyl-β-D-galactopyranoside (Magenta-Gal).

### Calculation and statistical analysis

The removal rates (%) of COD_Cr_, BOD, TN, SS, and VSS were calculated using Eq (1):

$$Removal\;rate\;(\%)=\frac{C_{inf}\;-\;C_{eff}}{C_{inf}}\times 100$$ (1)

where *C*_inf_ and *C*_eff_ represent the influent wastewater and treated effluent concentrations, respectively.

The log reduction values (LRVs) of *E*. *coli* and the potentially pathogenic bacterial groups were calculated using Eq (2):

$$LRV={log}_{10}\left(\frac{N_{inf}}{N_{eff}}\right)$$ (2)

where *N*_inf_ and *N*_eff_ are the abundances of *E*. *coli* (CFU mL^−1^) or a potentially pathogenic bacterial group (cells mL^−1^) in the influent wastewater and treated effluent, respectively.

Statistical differences in the average LRVs of the different WTFs were assessed using Student’s *t*-test with the Benjamini–Hochberg (BH) correction. Spearman’s rank correlation coefficient (*ρ*) calculation with the BH correction was performed to reveal the possible correlations among the abundances of *E*. *coli*, potentially pathogenic bacterial groups, and water quality parameters. The obtained quantitative data of *E*. *coli* CFU counts and potentially pathogenic bacterial abundances were log-transformed before the statistical analyses. Pooled data of all influent wastewater and treated effluent samples of WTFs were used to calculate the Spearman’s *ρ* values. Spearman’s *ρ* values were calculated using the corr.test function of the R package psych v.2.2.9 [60], and the Spearman’s *ρ* matrix was generated using the R package corr.plot v.0.92 [61]. Measurements below the LoQ were not included in the calculation. A *p*-value < 0.01 was considered significant. All statistical analyses were performed using R v.4.1.0 [62].

### Inclusivity in global research

Additional information regarding the ethical, cultural, and scientific considerations specific to inclusivity in global research is included in S1 Checklist.

## Results and discussion

### 16S rRNA gene amplicon sequencing analysis of influent and treated effluent wastewater samples

**Fig 1:**
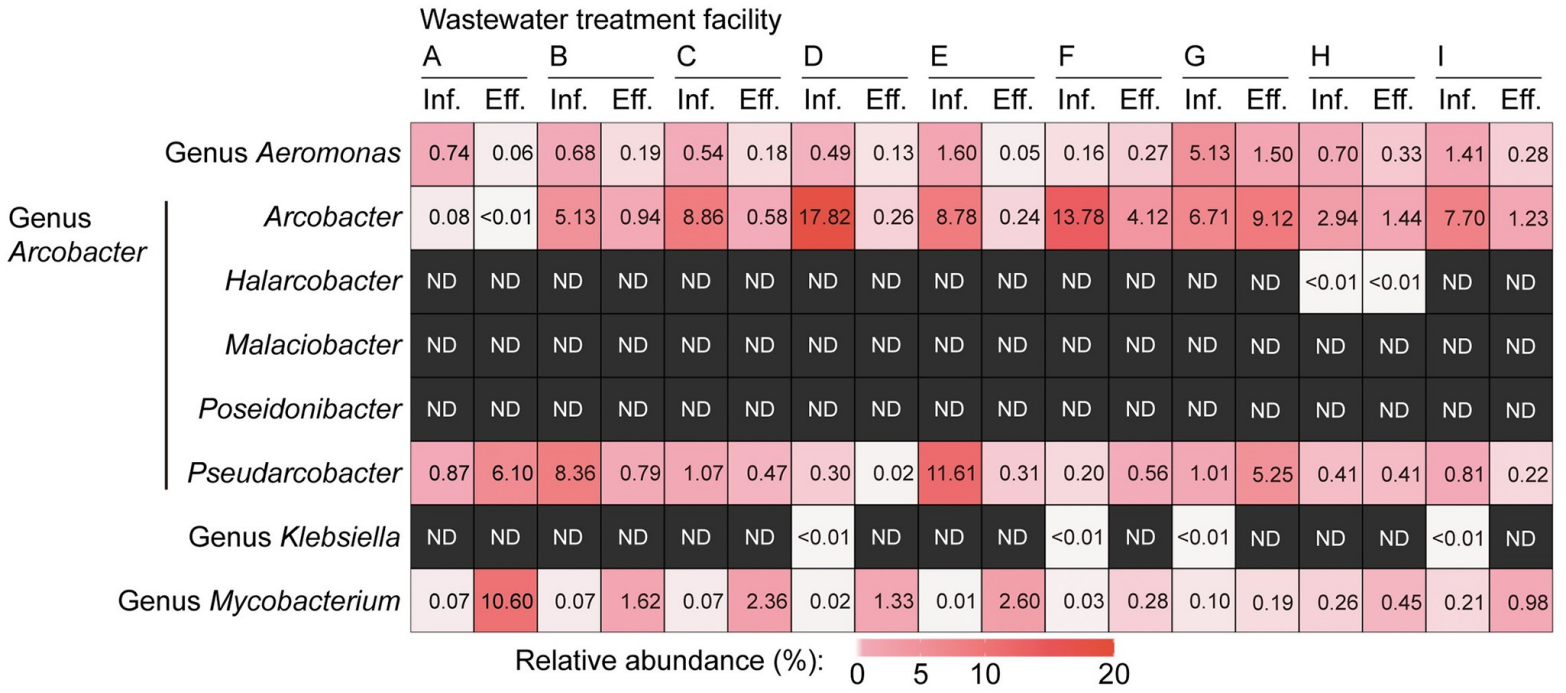
Relative 16S rRNA gene abundances of the genera *Aeromonas*, *Arcobacter*, *Klebsiella*, and *Mycobacterium* in the representative influent wastewater (inf.) and treated effluent (eff.) samples from the nine analyzed wastewater treatment facilities (A–I). ND, not detected.

16S rRNA gene amplicon sequencing analysis was performed to determine the presence and relative abundance of the targeted potentially pathogenic bacterial groups in the analyzed samples. In this study, 31,845–46,464 sequences of the 16S rRNA gene were obtained per sample from the influent wastewater and treated effluent samples following analysis using mothur. The relative 16S rRNA gene abundances of the genera *Aeromonas*, *Arcobacter*, *Klebsiella*, and *Mycobacterium* in the representative wastewater influent and treated effluent samples are summarized in Fig 1. The SILVA reference database (release 138.1) used in this study reflected the division of the genus *Arcobacter* into six *Arcobacteraceae* genera: *Aliarcobacter* (not defined in the reference database), *Pseudarcobacter*, *Malaciobacter*, *Halarcobacter*, *Poseidonibacter*, and *Arcobacter* (*sensu stricto*), as proposed by Pérez-Cataluña et al. [63]. However, On et al. [64] later proposed that these genera be considered a single genus *Arcobacter* based on phenotypic, genetic, and phylogenomic data. The genera *Aeromonas*, *Arcobacter*, and *Mycobacterium* were detected in all analyzed influent wastewater and treated effluent samples. The relative abundance of the genus *Arcobacter* (the sum of the relative abundances of the five above-mentioned *Arcobacteraceae* genera in the reference database) was markedly high (≥ 5.0%) in seven out of the nine analyzed samples, indicating the presence of *Arcobacter* as a dominant prokaryotic genus in most of the analyzed wastewater influent samples. The relatively high abundance of the genus *Arcobacter* in the influent wastewater prokaryotic communities is in good agreement with the previously reported 16S rRNA gene-targeted amplicon sequencing data [8–10]. Although the reason for the particularly high abundance of the genus *Arcobacter* in various influent wastewater samples is still under debate, a compelling explanation is their ability to grow in hydrogen sulfide-rich and oxygen-limited sewer pipe environments [65]. The relative abundances of the genus *Mycobacterium* in the effluent samples were consistently higher than those in the influent wastewater samples. The genus *Klebsiella*, which includes the KpSC, was extremely low in abundance or undetectable in most of the samples analyzed. This may have been caused by the insufficient variability of the 16S rRNA gene hypervariable region V4 to distinguish the *Enterobacteriaceae* genera [66].

**Fig 2:**
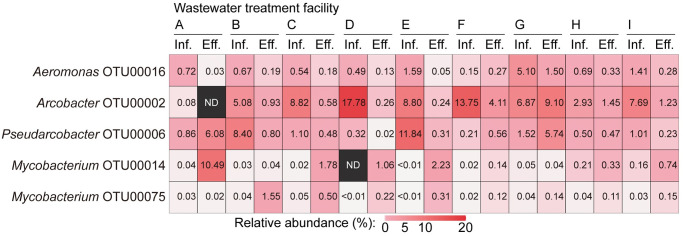
Relative abundances of dominant *Aeromonas*, *Arcobacter*, *Pseudarcobacter*, and *Mycobacterium* operational taxonomic units (OTUs) in the representative influent wastewater influent (inf.) and treated effluent (eff.) samples from the nine analyzed wastewater treatment facilities (A–I). ND, not detected.

The dominant *Aeromonas*, *Arcobacter*, *Pseudarcobacter*, and *Mycobacterium* OTUs with relative abundances greater than 1.0% in at least one of the analyzed samples are summarized in Fig 2. The genera *Aeromonas*, *Arcobacter*, and *Mycobacterium* were highly dominated by a few OTUs. The data suggest the presence of specific *Aeromonas*, *Arcobacter*, *Pseudarcobacter*, and *Mycobacterium* OTUs capable of occupying ecological niches in WTF environments. The metabolic flexibility of these OTUs might allow them to become dominant in highly dynamic/disturbed WTF environments [67]. The dominant *Aeromonas*, *Arcobacter*, and *Mycobacterium* OTUs (i.e., OTU00002, OTU00014, OTU00016, and OTU00075) have a high 16S rRNA gene sequence similarity (98.0–99.6%) to known biosafety level (BSL) 1 or 2 pathogenic bacterial species defined by the Japanese Society for Bacteriology [68] (Table 3). *Pseudarcobacter* OTU00006 showed relatively low 16S rRNA gene sequence similarity (94.9%) to pathogenic *Arcobacter* species (i.e., *Ar*. *butzleri* [BSL 1] and *Ar*. *cryaerophilus* [BSL 1]), but the sequence showed high similarity (98.0%) to *Ar*. *defluvii* with virulence traits [69]. The habitat preference of the dominant *Aeromonas*, *Arcobacter*, *Pseudarcobacter*, and *Mycobacterium* OTUs, as indicated by the ProkAtlas analysis, is summarized in Table 3. The data suggest that most of these OTUs can adapt to natural aquatic environments (e.g., fresh, ground, marine, and lake water), in addition to WTF environments. Therefore, the release of these potentially pathogenic OTUs into the environment without appropriate reduction may pose a potential threat to human health.

**Table 3:** Closest pathogenic bacterial relative(s) and habitat preferences of the dominant *Aeromonas*, *Arcobacter*, *Pseudarcobacter*, and *Mycobacterium* operational taxonomic units (OTUs) in the analyzed wastewater samples.

OTU ^a^	Closest pathogenic bacterial relative(s) (biosafety level) ^b^	Sequence similarity to the closest relative(s) (%)	Habitat preference (score) ^c^
*Aeromonas* OTU00016	*Aeromonas allosaccharophila* CECT 4199 (1)	98.8	Bioreactor (6.1), freshwater (5.4), groundwater (4.6), gut (6.0), marine (2.6), paper pulp (10.3), soil (5.3), subsurface (12.1), and wastewater (47.7)
	*Aeromonas caviae* CECT 838 (1)		
	*Aeromonas hydrophila* subsp. *hydrophila* ATCC 7966 (2)		
	*Aeromonas salmonicida* subsp. *achromogenes* NCIMB 1110 (1)		
	*Aeromonas salmonicida* subsp. *masoucida* NBRC 13784 (1)		
	*Aeromonas salmonicida* subsp. *pectinolytica* 34mel (1)		
	*Aeromonas salmonicida* subsp. *salmonicida* ATCC 33658 (1)		
	*Aeromonas veronii* CECT 4257 (2)		
	*Haemophilus piscium* CIP 106116 (1)		
*Arcobacter* OTU00002	*Arcobacter cryaerophilus* CCUG 17801 (1)	98.4	Freshwater (12.3), landfill (19.6), and wastewater (68.1)
*Pseudarcobacter* OTU00006	*Arcobacter butzleri* RM4018 (1)	94.9	Wastewater (100.0)
	*Arcobacter cryaerophilus* CCUG 17801 (1)		
*Mycobacterium* OTU00014	*Mycobacterium flavescens* ATCC 14474 (2)	98.0	Biofilm (16.0), freshwater (8.6), fungus (2.1), lake water (24.3), marine (5.6), peat (1.7), rhizosphere (16.6), root (3.1), soil (20.0), and subsurface (2.2)
*Mycobacterium* OTU00075	*Mycobacterium sphagni* ATCC 33027 (2)	99.6	Activated sludge (1.6), biofilm (11.5), freshwater (6.0), freshwater sediment (2.7), fungus (5.6), lake water (6.7), marine (7.6), peat (3.1), rhizosphere (20.7), root (2.8), soil (30.4), and wetland (1.4)
	*Mycobacterium brisbanense* ATCC 49938 (1)		

^a^ The consensus taxonomy for each OTU was based on the SILVA reference database (release 138.1) [48].

^b^ Pathogenic bacterial species were defined by the Japanese Society of Bacteriology [68].

^c^ The score represents the compositions of the retrieved environmental categories through the ProkAtlas analysis [56].

### PCR primers for quantitative detection of *Aeromonas*

16S rRNA gene-targeted PCR primers for the amplification of *Aeromonas* sequences have been reported previously [70, 71]. However, our analysis revealed that the *in silico* coverage of previously reported *Aeromonas*-specific primer sets A16SF/A16SR and Aer66f/Aer613r in the SILVA reference database (release 138.1) (sequence collection: RefNR) was relatively low (66.9% [658/983 sequences] and 69.0% [644/933 sequences] of coverage at 0 mismatch, respectively) (S2 Table). Therefore, for a more comprehensive detection of environmental *Aeromonas*, we designed a new PCR primer set (Aero581F/Aero848R), which showed a slightly improved *in silico* coverage of 74.3% (765/1030 sequences) at 0 mismatches in the reference database (sequence collection: RefNR). In addition, the new PCR primer set was shown to cover 32 out of the 35 eligible 16S rRNA gene sequences of *Aeromonas* type strains in the reference database (sequence collection: LTP) without any mismatches. Therefore, the new primer set is expected to detect a broader range of environmental *Aeromonas* than the previously reported primer sets. Moreover, the relatively short amplicon size of Aero581F/Aero848R (268 bp) is advantageous to ensure efficient amplification. The LoD and LoQ values of the newly developed *Aeromonas* qPCR assay were 1 and 10 copies reaction^−1^, respectively. Therefore, although previously reported PCR primer sets have been successfully applied to the monitoring of environmental *Aeromonas* [71, 72], the new qPCR assay described herein is an effective alternative option for the quantitative detection of environmental *Aeromonas*.

### Quantitative detection and reduction of *E*. *coli* and potentially pathogenic bacterial groups in existing WTFs

**Fig 3:**
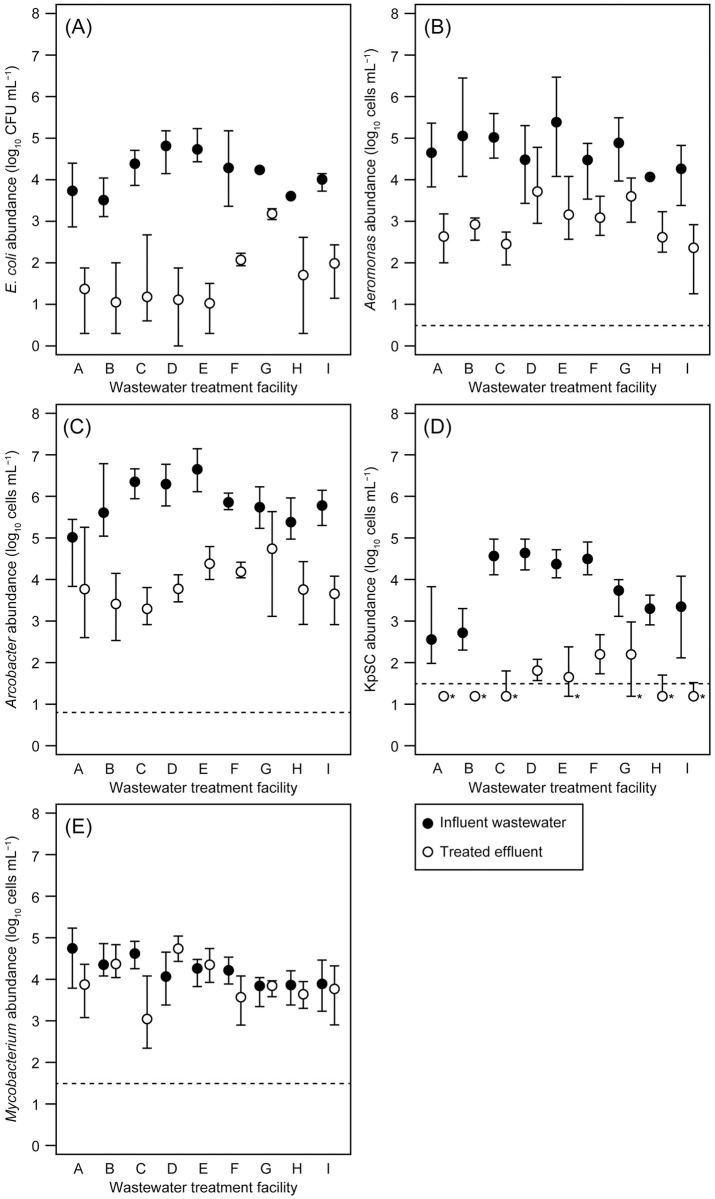
Geometric mean abundances of (A) *Escherichia coli*, (B) *Aeromonas*, (C) *Arcobacter*, (D) *Klebsiella pneumoniae* species complex (KpSC), and (E) *Mycobacterium* in the influent wastewater and treated effluent of the nine analyzed wastewater treatment facilities (A–I). Error bars indicate the maximum and minimum abundances. Horizontal dashed lines indicate the limit of quantification (LoQ) for each quantitative real-time polymerase chain reaction assay. *, KpSC abundances below the LoQ are indicated by the value of LoQ/2.

**Fig 4:**
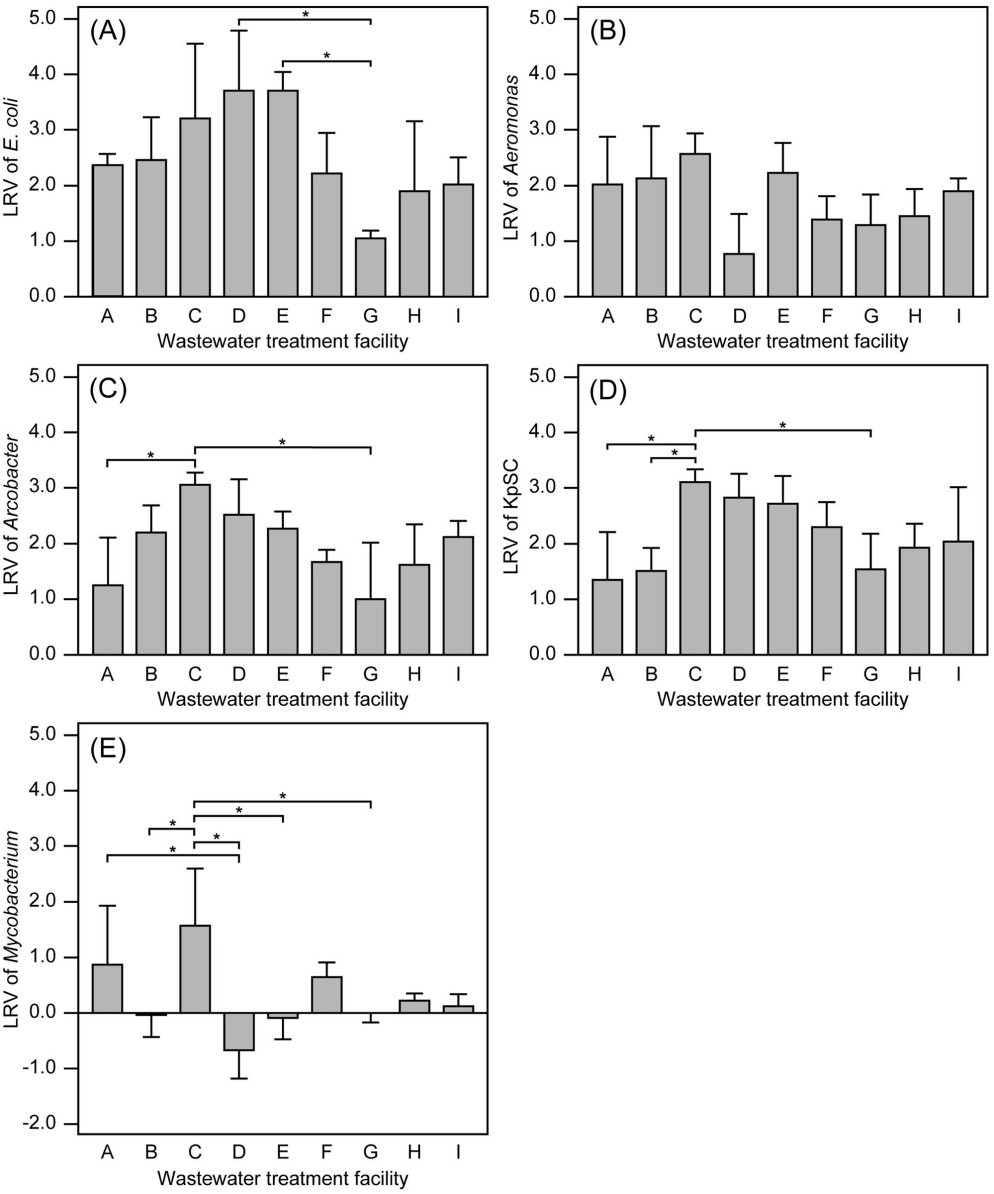
Average log reduction values (LRVs) of (A) *Escherichia coli*, (B) *Aeromonas*, (C) *Arcobacter*, (D) *Klebsiella pneumoniae* species complex (KpSC), and (E) *Mycobacterium* in the nine analyzed wastewater treatment facilities (A–I). Error bars indicate the standard deviation. *, *p* < 0.01 according to Student’s *t*-test with the Benjamini–Hochberg correction.

The abundances of selected potentially pathogenic bacterial groups of *Aeromonas*, *Arcobacter*, KpSC, and *Mycobacterium* in the influent wastewater and treated effluent of nine WTFs were determined using qPCR assays. All target bacterial groups were detected with varying concentrations in all influent wastewater samples. The abundances of *Aeromonas*, *Arcobacter*, and *Mycobacterium* could be quantified in all analyzed effluent samples. In contrast, KpSC was not detected or was below the LoQ (i.e., 3.1 × 10^1^ cells mL^−1^) in 17 out of 34 analyzed effluent samples. The geometric mean abundances of *E*. *coli* (determined using the Compact Dry “Nissui” EC method) and potentially pathogenic bacterial groups (determined using qPCR assays) in the influent wastewater and treated effluent of the WTFs are summarized in Fig 3. The average LRVs of *E*. *coli* and potentially pathogenic bacterial groups in the WTFs are summarized in Fig 4.

#### Quantitative data on *E*. *coli*

The geometric mean abundances of *E*. *coli* in the influent wastewater and treated effluent samples were 3.2 × 10^3^ to 6.5 × 10^4^ and 1.1 × 10^1^ to 1.5 × 10^3^ (CFU mL^−1^), respectively (Fig 3A). The geometric mean *E*. *coli* abundances in the treated effluents were slightly or markedly higher than the WHO’s strictest guideline value for unrestricted irrigation (1,000 CFU 100 mL^−1^) [34]. In contrast, the geometric mean *E*. *coli* abundances in the treated effluent of seven WTFs were below the WHO’s guideline value for restricted agricultural irrigation for processed food crops (10,000 CFU 100 mL^−1^) [34]. Although the treated effluent from Bangkok’s domestic WTFs is used for street washing, irrigation of green belts, cleaning of temples, etc., without the application of a disinfection process [44], the *E*. *coli* abundance data suggest that the hygienic safety of the treated effluent, especially that from centralized domestic WTF G, should be rigorously evaluated. Notably, fecal indicators, including *E*. *coli*, have not been used in any effluent quality standards in Thailand [73]. The average LRVs of *E*. *coli* calculated from the obtained CFU counts ranged from 1.05 to 3.71 (Fig 4A). A significant difference in average *E*. *coli* LRVs was observed only between WTF G (1.05) and WTFs D (3.71, *p* = 0.0019) or E (3.71, *p* = 0.0019). The LRVs were consistent with those of several previously reported WTFs [1, 74, 75]. The markedly high geometric mean *E*. *coli* abundance found in the effluent of centralized domestic WTF G (i.e., 1.5 × 10^3^ CFU mL^−1^) is likely attributed to the low LRV (1.05).

#### Quantitative data on *Aeromonas*

The geometric mean abundance of *Aeromonas* in the influent wastewater samples was 1.2 × 10^4^ to 2.4 × 10^5^ cells mL^−1^, whereas that in the treated effluent was 2.3 × 10^2^ to 5.2 × 10^3^ cells mL^−1^ (Fig 3B). The relatively high *Aeromonas* abundances in the influent wastewater and treated effluent are consistent with those reported in previous studies. Martone-Rocha et al. detected *Aeromonas* in the raw sewage influent of a Brazilian sanitary sewage stabilization pond treatment system, with abundance fluctuations ranging from <3 to 3.0 × 10^9^ most probable number 100 mL^−1^ [18]. Moreover, in a Moroccan basin wastewater treatment system, the average *Aeromonas* abundances in the influent wastewater and treated effluent were 3.3 × 10^4^ and 2.4 × 10^3^ CFU mL^−1^, respectively [17]. The average LRVs of *Aeromonas* in the nine WTFs analyzed in this study ranged from 0.77 to 2.57 (Fig 4B), and no significant difference was found in the average LRVs (*p* = 0.015–0.90). Most of the average LRVs of *Aeromonas* were almost comparable with those of a Brazilian sanitary sewage stabilization pond (2.57) [18] and a Moroccan basin wastewater treatment system (1.14) [17].

#### Quantitative data on *Arcobacter*

Among the quantified potentially pathogenic bacterial groups, particularly high abundances of *Arcobacter* were confirmed in the analyzed influent wastewater and treated effluent samples (Fig 3C). The geometric mean abundances of *Arcobacter* in the influent wastewater ranged from 1.0 × 10^5^ to 4.5 × 10^6^ cells mL^−1^. The geometric mean abundances in the treated effluent ranged from 2.0 × 10^3^ to 5.5 × 10^4^ cells mL^−1^. The primer set Arco605F/Arco688R [40] used in this study was developed for the quantitative detection of members of the genus *Arcobacter* as proposed by On et al. [64]. Highly abundant *Arcobacter* populations in wastewater treatment environments have also been reported in previous research. Yang et al. [12] reported that the 23S rRNA gene abundances of *Arcobacter* in the influent and treated effluent of a rural domestic WTF with a membrane bioreactor located in Beijing, China were 1.01 × 10^11^ and 1.45 × 10^8^ copies L^−1^, respectively (equivalent to 2.02 × 10^7^ and 2.90 × 10^4^ cells mL^−1^, respectively). Fluorescence *in situ* hybridization-based quantification by Kristensen et al. [9] showed that the range of *Arcobacter* abundance in the influent wastewater of 14 full-scale Danish municipal WTFs was 4.2 × 10^5^ to 3.2 × 10^7^ cells mL^−1^, whereas that in the treated effluent was 3.5 × 10^4^ to 5.1 × 10^6^ cells mL^−1^. Ghaju Shrestha et al. [22] reported the 16S rRNA gene copy numbers of *Arcobacter* in two WTFs in southern Arizona using conventional activated sludge and biological trickling filter processes, respectively. The average abundances of the influent *Arcobacter* 16S rRNA gene in the WTFs were 7.9 and 8.5 log_10_ copies L^−1^ (equivalent to 1.6 × 10^4^ and 6.3 × 10^4^ cells mL^−1^, respectively), whereas those in the treated effluent were 6.4 and 6.1 log_10_ copies L^−1^ (equivalent to 5.0 × 10^2^ and 2.5 × 10^2^ cells mL^−1^, respectively). The observed average LRVs of *Arcobacter* in the nine WTFs analyzed in this study were between 1.00 and 3.06 (Fig 4C). The maximum average LRV of 3.06 was observed in WTF C, which is significantly higher than those observed at WTFs A (1.25, *p* = 0.0049) and G (1.00, *p* = 0.0023). The observed average LRVs were comparable to or slightly lower than those of a full-scale Danish municipal WTF with an enhanced biological phosphorus removal process (LRV: 2.40) [9], two WTFs in southern Arizona (1.7 [conventional activated sludge process] and 2.3 [biological trickling filter process]) [22], and a rural Chinese domestic WTF with a membrane bioreactor (2.84) [12].

#### Quantitative data on KpSC

The geometric mean abundances of KpSC in the influent wastewater and treated effluent are shown in (Fig 3D). The geometric mean abundances of KpSC in the influent wastewater ranged from 3.6 × 10^2^ to 4.3 × 10^4^ cells mL^−1^. The geometric mean abundances of KpSC in the treated effluent were comparatively lower than that of the other quantified potentially pathogenic bacterial groups: the geometric mean abundances ranged from < 3.1 × 10^1^ (below the LoQ) to 1.6 × 10^2^ cells mL^−1^. Since the highly specific qPCR assay for KpSC quantification was not available prior to the report by Barbier et al. [27], reliable data on KpSC abundance in the environment had not been obtained. To the best of our knowledge, this is the first report on the KpSC abundances in the influent wastewater and treated effluent of existing WTFs obtained using the specific qPCR assay. The observed average LRVs of KpSC in the analyzed WTFs ranged from 1.35 to 3.11 (Fig 4D). The maximum LRV of 3.11 was observed in WTF C, which was significantly higher than those of WTFs A (1.35, *p* = 0.0082), B (1.51, *p* = 0.0088), and G (1.54, *p* = 0.0088).

#### Quantitative data on *Mycobacterium*

The geometric mean abundances of *Mycobacterium* in the influent wastewater and treated effluent of the existing WTFs are shown in Fig 3E. In contrast to the other quantified potentially pathogenic bacterial groups, similar abundance levels were confirmed in numerous analyzed influent wastewater and treated effluent samples. The maximum and minimum geometric mean abundances of *Mycobacterium* in the influent wastewater were 5.5 × 10^4^ and 6.9 × 10^3^ cells mL^−1^, respectively. The maximum and minimum geometric mean abundances of *Mycobacterium* in the treated effluent were 5.5 × 10^4^ and 1.1 × 10^3^ cells mL^−1^, respectively. The *Mycobacterium* abundances of influent wastewater and treated effluent samples were higher than those of a WTF located in Paris that used a combined process of physical-chemical decantation for suspended matter and phosphorus removal, followed by biofiltration for organic matter and nitrogen removal [33]. The average *Mycobacterium* 16S rRNA gene abundance in the untreated influent sewage of the WTF in Paris was 5.5 × 10^5^ copies L^−1^ (equivalent to 5.5 × 10^2^ cells mL^−1^), whereas the *Mycobacterium* 16S rRNA gene was not detected in the treated effluent. The LRVs of *Mycobacterium* in the WTFs analyzed in this study are shown in Fig 4E. The LRVs of *Mycobacterium* were lower than that of other quantified potentially pathogenic bacterial groups: the average LRVs ranged from −0.67 to 1.57. The negative average LRVs of *Mycobacterium* suggested the growth of *Mycobacterium* during wastewater treatment in the WTFs. The growth of *Mycobacterium* in the WTFs analyzed in this study is not surprising as *Mycobacterium* has been reported as an abundant bacterial genus in the activated sludge of many biological WTFs [76, 77]. Moreover, the growth of *Mycobacterium* in various WTFs, including those investigated in this study, might be due to their ability to degrade cholesterol, a major component of sewage [77]. The highest average LRV of 1.57 was observed in WTF C, which was significantly higher than those observed in WTFs B (−0.018, *p* = 0.0076), D (−0.67, *p* = 0.00049), E (−0.088, *p* = 0.0076), and G (−0.002, *p* = 0.0076). The second highest average LRV was observed in WTF A (0.87), which was significantly higher than that of WTF D (−0.67, *p* = 0.0076). The average LRVs observed in all WTFs investigated in this study were lower than that of a WTF in Paris using a consecutive biofiltration process (average LRV: 3.2) [33].

#### Summary of the obtained quantitative data on *E*. *coli* and potentially pathogenic bacterial groups

In this study, one or more average LRVs of *Aeromonas*, *Arcobacter*, and KpSC were confirmed in all or most of the WTFs analyzed. In contrast, the analyzed WTFs did not effectively remove the genus *Mycobacterium*. Despite differences in the treatment scale, treatment system, and operating environment, most analyzed WTFs showed a similar reduction potential for the quantified potentially pathogenic bacterial groups. The significant differences in the LRVs of *Arcobacter*, KpSC, and *Mycobacterium* observed between some WTFs may be attributed to the significant differences in the water quality parameters of the influent wastewater and treated effluent, as discussed later in this paper. The changes in the water quality parameters may be influenced by the configuration and operating conditions of the WTFs. Among the various treatment systems analyzed in this study, the combined system of an anaerobic filter bed and contact aeration used in rural community WTF C showed the highest average LRVs for all potentially pathogenic bacterial groups quantified. The data suggest that the combined system is an appropriate option for reducing the health risks caused by *Aeromonas*, *Arcobacter*, KpSC, and *Mycobacterium* in wastewater. In contrast, the successful quantification of *Aeromonas*, *Arcobacter*, KpSC, and *Mycobacterium* in treated effluents suggests the need for disinfection prior to discharge into the environment and reuse for human purposes.

### Correlations between potentially pathogenic bacterial groups and *E*. *coli*

**Fig 5:**
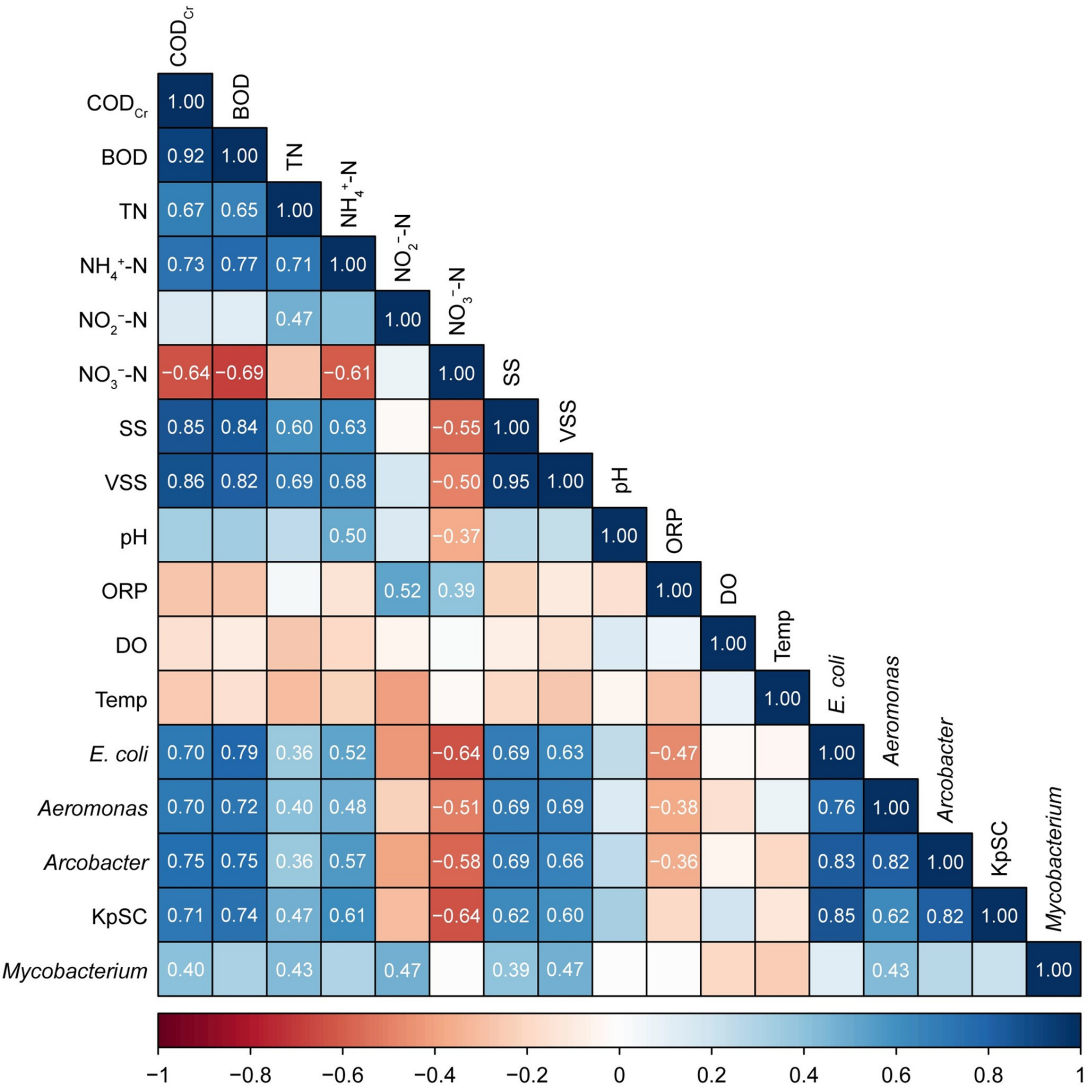
Spearman’s rank correlation coefficients between the abundances of *Escherichia coli* and potentially pathogenic bacterial groups and water quality parameters. Spearman’s rank correlation coefficients are shown only for *p* < 0.01 with the Benjamini–Hochberg correction. COD_Cr_: chemical oxygen demand concentration determined by the potassium dichromate method (mg L^−1^), BOD: biochemical oxygen demand concentration (mg L^−1^), TN: total nitrogen concentration (mg L^−1^), NH_4_^+^-N: ammonium-nitrogen concentration (mg L^−1^), NO_2_^−^-N: nitrite-nitrogen concentration (mg L^−1^), NO_3_^−^-N: nitrate-nitrogen concentration (mg L^−1^), SS: suspended solids concentration (mg L^−1^), VSS: volatile suspended solids concentration (mg L^−1^), ORP: oxidation-reduction potential (mV), DO: dissolved oxygen concentration (mg L^−1^), Temp: water temperature (°C), *E*. *coli*: *Escherichia coli* abundance (log_10_ colony-forming units mL^−1^), *Aeromonas*: *Aeromonas* abundance (log_10_ cells mL^−1^), *Arcobacter*: *Arcobacter* abundance (log_10_ cells mL^−1^), KpSC: *Klebsiella pneumoniae* species complex abundance (log_10_ cells mL^−1^), and *Mycobacterium*: *Mycobacterium* abundance (log_10_ cells mL^−1^).

**Fig 6:**
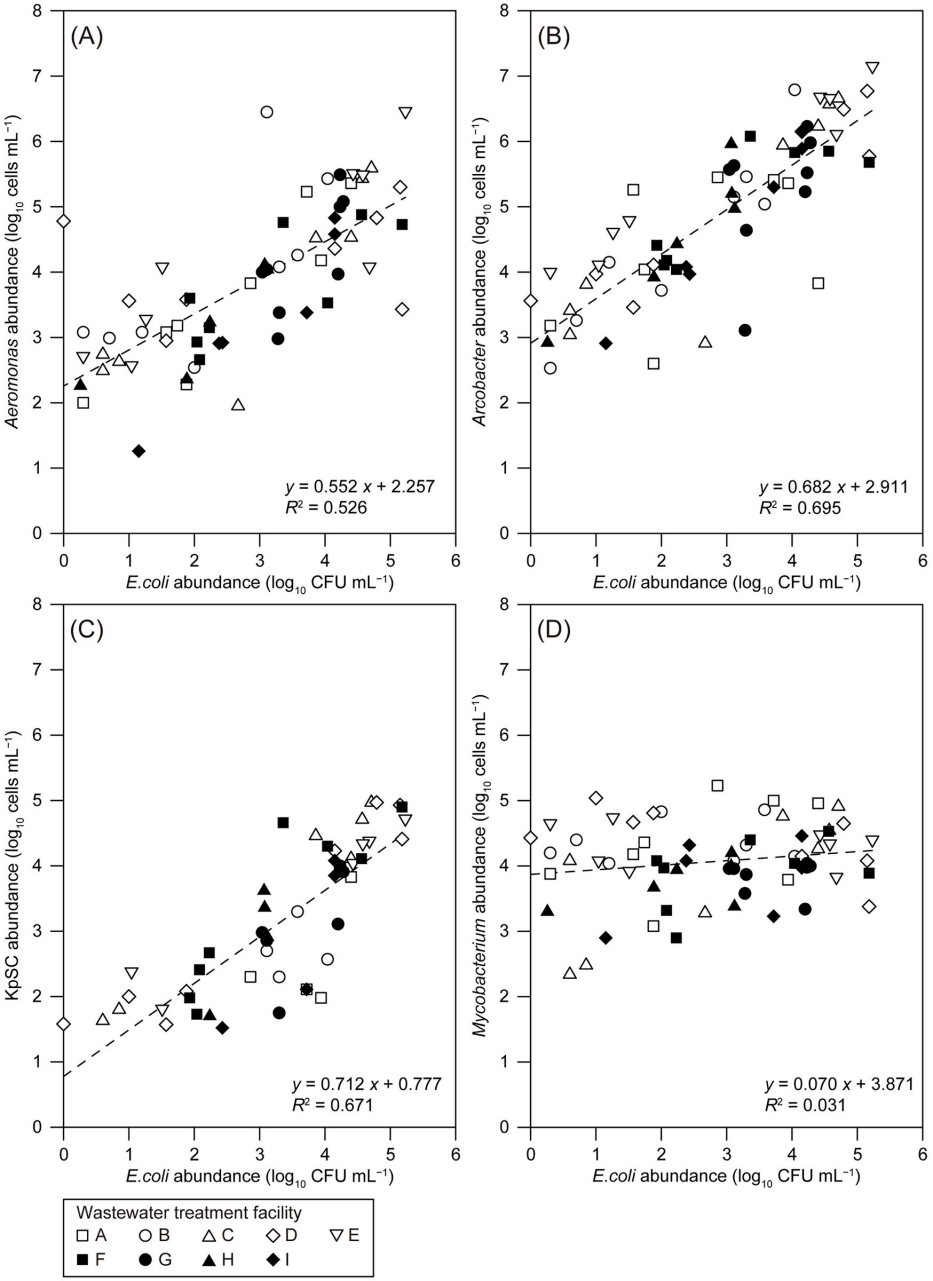
Correlations between the abundances of *Escherichia coli* (log_10_ colony-forming units [CFU] mL^−^1) and (A) *Aeromonas*, (B) *Arcobacter*, (C) *Klebsiella pneumoniae* species complex (KpSC), or (D) *Mycobacterium* (log_10_ cells mL^−^1) in the wastewater treatment facilities.

Correlations between the abundances of *E*. *coli* and the quantified potentially pathogenic bacterial groups are shown in Figs 5 and 6. Based on the Spearman’s *ρ* values, there was a significant positive correlation between the abundances of *E*. *coli*, *Aeromonas*, *Arcobacter*, and KpSC (*ρ* = 0.62–0.85, *p* = 0.00) (Fig 5). In addition, a linear positive correlation was found between the abundances of *E*. *coli* and *Aeromonas*, *Arcobacter*, or KpSC (coefficient of determination *R*^2^ = 0.526–0.695) (Fig 6A–6C). These data suggest the applicability of *E*. *coli* as a bacterial indicator of *Aeromonas*, *Arcobacter*, and KpSC contamination levels in wastewater-related samples. Correlations between the abundances of *E*. *coli* and *Aeromonas* or *Arcobacter* have also been reported in some non-wastewater environments. Solaiman et al. reported a positive correlation between the abundances of *Aeromonas* and *E*. *coli* in reclaimed water sources and non-tidal freshwater rivers/creeks, whereas no correlation was found in tidal brackish water and irrigation ponds [78]. A positive correlation between *E*. *coli* and *Arcobacter* abundances in surface water samples from canals and the Chao Phraya River in Bangkok, Thailand, was also confirmed in our previous report [40]. The insignificant correlation between *E*. *coli* and *Mycobacterium* abundances (Figs 5 and 6D) also indicates the possible importance of independent monitoring of *Mycobacterium*.

### Correlation between abundances of potentially pathogenic bacterial groups and water quality parameters

The water quality parameters of the influent wastewater and treated effluent of the WTFs and the removal rates (%) of COD_Cr_, BOD, TN, SS, and VSS are summarized in S3 Table. The Spearman’s *ρ* values between the quantified potentially pathogenic bacterial groups and the water quality parameters are shown in Fig 5. The Spearman’s *ρ* values indicated the presence of positive or negative correlations between the abundances of potentially pathogenic bacterial groups and several water quality parameters. *Aeromonas* and *Arcobacter* abundances were positively correlated with the COD_Cr_, BOD, TN, NH_4_^+^-N, SS, and VSS concentrations (*ρ* = 0.36–0.75, *p* = 0.00) but negatively correlated with the NO_3_^−^-N concentration and ORP (*ρ* = −0.58 to −0.36, *p* = 0.00). Similarly, KpSC abundance was positively correlated with the COD_Cr_, BOD, TN, NH_4_^+^-N, SS, and VSS concentrations (*ρ* = 0.47–0.74, *p* = 0.00), whereas it was negatively correlated only with the NO_3_^−^-N concentration (*ρ* = −0.64, *p* = 0.00). *Mycobacterium* abundance was significantly positively correlated with COD_Cr_, TN, NO_2_^−^-N, SS, and VSS (*ρ* = 0.39–047, *p* = 0.00), but no significant negative correlation was found with the other water quality parameters (*p* = 0.10 and 0.04 for the DO concentration and water temperature, respectively).

Pathogen reduction in WTFs is achieved by a combination of physical, chemical, and biological mechanisms [75]. The correlation between the four quantified potentially pathogenic bacterial groups with the SS and VSS concentrations suggests that the adsorption of these bacterial cells to the activated sludge and their subsequent removal by the excess sludge is one of the mechanisms to reduce the abundance of these potentially pathogenic bacterial groups [9]. Radomski et al. also reported that the *Mycobacterium* reduction in a WTF was related to the behaviors of insoluble compounds [33]. However, relatively high geometric mean abundances of *Aeromonas*, *Arcobacter*, and *Mycobacterium* (≥ 10^2^ cells mL^−1^) were observed in the analyzed effluent samples with relatively low average SS and VSS concentrations (2–19 and 1–16 mg L^−1^, respectively). These data suggest that a fraction of these potentially pathogenic bacterial cells do not flocculate and attach well to the sludge flocs, which is consistent with the report by Kristensen et al. [9], who studied the abundance and reduction of *Arcobacter* in full-scale municipal WTFs.

The correlations of *Aeromonas*, *Arcobacter*, and KpSC abundances with COD_Cr_ and BOD concentrations may reflect their robust growth/survival potential under organic matter-rich wastewater conditions. In contrast, relatively low or insignificant correlations were found between *Mycobacterium* abundance and COD_Cr_ or BOD concentrations, respectively. This may be explained by the superior growth/survival potential of *Mycobacterium* under oligotrophic conditions [79].

Based on the correlations between the concentrations of NO_3_^−^-N, NH_4_^+^-N, and ORP with the abundances of the potentially pathogenic bacterial groups, *Aeromonas*, *Arcobacter*, and KpSC are more susceptible to oxidative conditions, where NO_3_^−^-N can be accumulated by the biological nitrification of NH_4_^+^-N, than *Mycobacterium*. Therefore, improving the nitrification capacity of WTFs may be effective in removing *Aeromonas*, *Arcobacter*, and KpSC. The observed correlation between the TN concentration and abundance of potentially pathogenic bacterial groups, including *Mycobacterium*, might be caused by the enhanced survival potential of the potentially pathogenic bacterial groups in the presence of the available TN [75]. Considering this correlation, the enhancement or introduction of a biological denitrification process in WTFs could ensure further reduction of *Aeromonas*, *Arcobacter*, and KpSC, as well as the effective reduction of *Mycobacterium*. Tomioka et al. [40] reported a negative correlation between *Arcobacter* abundance and NO_3_^−^-N concentration or ORP, and a positive correlation between *Arcobacter* abundance and TN or NH_4_^+^-N concentrations in surface water samples from canals and the Chao Phraya River in Bangkok, Thailand. In our study, a significant correlation between the NO_2_^−^-N concentration and quantified bacterial group abundance was found only in *Mycobacterium*. This correlation suggests the superior growth/survival capacity of *Mycobacterium* under nitrite-accumulating conditions, where incomplete nitrification occurs by insufficient oxygen supply. In fact, adaptation to transient and prolonged oxygen deprivation has been demonstrated in saprophytic and pathogenic *Mycobacterium* species [80].

Overall, our data indicate that changes in several water quality parameters affect the abundances of the potentially pathogenic bacterial groups of *Aeromonas*, *Arcobacter*, KpSC, and *Mycobacterium*. The data also suggest that WTFs with a superior removal capacity of organic matter, nitrogen compounds (caused by biological nitrification and subsequent denitrification), and suspended solids can effectively remove the potentially pathogenic bacteria. Of note, rural community WTF C with a high average removal rate of COD_Cr_ (93%), BOD (94%), TN (77%), SS (98%), and VSS (98%) (S3 Table) showed the highest average LRVs of *Aeromonas* (2.57), *Arcobacter* (3.06), KpSC (3.11), and *Mycobacterium* (1.57). Also, although similar levels of *Aeromonas*, *Arcobacter*, and KpSC reduction were confirmed in WTFs C and D (*p* = 0.015–0.61), the average LRV of *Mycobacterium* in the combined anaerobic filter bed and contact aeration system-based WTF C (1.56) was significantly higher than that of the contact aeration system-based WTF D (−0.67, *p* = 0.00049) (Fig 4). The effective *Mycobacterium* reduction by WTF C might be because of efficient TN removal (average removal rate: 77%) via the anaerobic filter bed process in which biological denitrification occurs effectively. This study has some limitations. Conventional qPCR assays cannot distinguish between viable and non-viable cells, which pose a low health risk. Therefore, further viability-qPCR assay-based surveys [81] may provide additional insights into the development and appropriate management of WTFs to produce safe and hygienic water. In addition, the correlation between antibiotic-resistant bacteria, antibiotic-resistance genes, and water quality parameters should be further investigated to comprehensively reduce health risks associated with the discharge and use of treated wastewater [82]. Furthermore, certain *Aeromonas*, *Arcobacter*, and *Mycobacterium* members can be highly resistant to chlorine disinfection, which is the most cost-effective disinfection method [83]. Therefore, the efficacy of different disinfection methods against these potentially pathogenic bacterial groups warrants further investigation.

## Conclusions

In this study, we successfully determined the abundance of potentially pathogenic bacterial groups *Aeromonas*, *Arcobacter*, KpSC, and *Mycobacterium* in the influent wastewater and treated effluents of nine existing WTFs located in Japan and Thailand using qPCR assays. One or more average LRVs of *Aeromonas*, *Arcobacter*, and KpSC were confirmed in most of the WTFs analyzed. However, owing to the relatively high geometric mean abundances of *Aeromonas*, *Arcobacter*, and *Mycobacterium* (≥ 10^4^ cells mL^−1^) in the influent wastewater and the relatively low LRV of *Mycobacterium* (ranging from −0.67 to 1.57) in the existing WTFs, high geometric mean abundances of *Aeromonas*, *Arcobacter*, and *Mycobacterium* (≥ 10^2^ cells mL^−1^) were detected in all treated effluent samples analyzed. The LRVs of *Arcobacter*, KpSC, and *Mycobacterium* in some WTFs differed significantly. A linear, positive correlation was found between the abundances of *E*. *coli* and *Aeromonas*, *Arcobacter*, or KpSC, suggesting the applicability of *E*. *coli* as a bacterial indicator to predict the contamination levels of *Aeromonas*, *Arcobacter*, and KpSC. Finally, Spearman’s *ρ* values suggested that changes in several water quality parameters (COD_Cr_, BOD, TN, NH_4_^+^-N, NO_2_^−^-N, NO_3_^−^-N, SS, VSS, and ORP) affected the abundance of the potentially pathogenic bacterial groups. The data obtained in this study provide new insights into the establishment and proper administration of WTFs to effectively generate safe hygienic water.

## Supporting information

S1 Checklist Additional information regarding the ethical, cultural, and scientific considerations specific to inclusivity in global research. (PDF)

S1 Text Detailed methods of quantitative real-time polymerase chain reaction (qPCR) assays. (PDF)

S1 Table Sequences of synthetic standard DNA fragments used for quantitative real-time polymerase chain reaction assays. (PDF)

S2 Table *In silico* coverage and specificity of primer sets targeting the 16S rRNA gene of the genus *Aeromonas*. (PDF)

S3 Table Overview of the water quality parameters and removal rates of COD_Cr_, BOD, TN, SS, and VSS in the wastewater treatment facilities analyzed in this study (average ± standard deviation). (PDF)

## References

[1] Hube S, Wu B. Mitigation of emerging pollutants and pathogens in decentralized wastewater treatment processes: A review. Sci. Total Environ. 2021;779:146545. doi: 10.1016/j.scitotenv.2021.146545 33752021

[2] Levine AD, Asano T. Recovering sustainable water from wastewater. Environ. Sci. Technol. 2004;38(11):201A–208A. doi: 10.1021/es040504n 15224722

[3] Jones ER, van Vliet MTH, Qadir M, Bierkens MFP. Country-level and gridded estimates of wastewater production, collection, treatment and reuse. Earth Syst. Sci. Data 2021;13(2):237–254. doi: 10.5194/essd-13-237-2021

[4] Naidoo S, Olaniran AO. Treated wastewater effluent as a source of microbial pollution of surface water resources. Int. J. Environ. Res. Public Health 2014;11(1):249–270. doi: 10.3390/ijerph110100249 24366046 PMC3924443

[5] Sun Y-X, Wu Q-Y, Hu H-Y, Tian J. Effect of ammonia on the formation of THMs and HAAs in secondary effluent chlorination. Chemosphere 2009(5);76:631–637. doi: 10.1016/j.chemosphere.2009.04.041 19482329

[6] Delanka-Pedige HMK, Munasinghe-Arachchige SP, Zhang Y, Nirmalakhandan N. Bacteria and virus reduction in secondary treatment: Potential for minimizing post disinfectant demand. Water Res. 2020;177:115802. doi: 10.1016/j.watres.2020.115802 32311576

[7] Cai L, Zhang T. Detecting human bacterial pathogens in wastewater treatment plants by a high-throughput shotgun sequencing technique. Environ. Sci. Technol. 2013;47(10):5433–5441. doi: 10.1021/es400275r 23594284

[8] Numberger D, Ganzert L, Zoccarato L, Mühldorfer K, Sauer S, Grossart H-P, et al. Characterization of bacterial communities in wastewater with enhanced taxonomic resolution by full-length 16S rRNA sequencing. Sci. Rep. 2019;9:9673. doi: 10.1038/s41598-019-46015-z 31273307 PMC6609626

[9] Kristensen JM, Nierychlo M, Albertsen M, Nielsen PH. Bacteria from the genus *Arcobacter* are abundant in effluent from wastewater treatment plants. Appl. Environ. Microbiol. 2020;86(9):e03044–19. doi: 10.1128/AEM.03044-19 32111585 PMC7170470

[10] Galagoda R, Chanto M, Takemura Y, Tomioka N, Syutsubo K, Honda R, et al. Quantitative 16S rRNA gene amplicon sequencing for comprehensive pathogenic bacterial tracking in a municipal wastewater treatment plant. ACS EST Water 2023;3(4), 923–933. doi: 10.1021/acsestwater.2c00349

[11] Nnadozie CF, Kumari S, Bux F. Status of pathogens, antibiotic resistance genes and antibiotic residues in wastewater treatment systems. Rev. Environ. Sci. Biotechnol. 2017;16:491–515. doi: 10.1007/s11157-017-9438-x

[12] Yang B, Kong X, Cui B, Jin D, Deng Y, Zhuang X, et al. Impact of rural domestic wastewater irrigation on the physicochemical and microbiological properties of pakchoi and soil. Water 2015;7(5):1825–1839. doi: 10.3390/w7051825

[13] Fernández-Bravo A, Figueras MJ. An update on the genus Aeromonas: Taxonomy, epidemiology, and pathogenicity. Microorganisms 2020;8(1):129. doi: 10.3390/microorganisms8010129 31963469 PMC7022790

[14] Janda JM, Abbott SL. The genus *Aeromonas*: Taxonomy, pathogenicity, and infection. Clin. Microbiol. Rev. 2010;23(1):35–73. doi: 10.1128/CMR.00039-09 20065325 PMC2806660

[15] Lamy B, Kodjo A, colBVH Study Group, Laurent F. Prospective nationwide study of *Aeromonas* infections in France. J. Clin. Microbiol. 2009;47(4):1234–1237. doi: 10.1128/JCM.00155-09 19244464 PMC2668343

[16] Chen P-L, Lamy B, Ko W-C. *Aeromonas dhakensis*, an increasingly recognized human pathogen. Front. Microbiol. 2016;7:793. doi: 10.3389/fmicb.2016.00793 27303382 PMC4882333

[17] Boussaid A, Baleux B, Hassani L, Lesne J. *Aeromonas* species in stabilization ponds in the arid region of Marrakesh, Morocco, and relation to fecal-pollution and climatic factors. Microb. Ecol. 1991;21:11–20. doi: 10.1007/BF02539141 24194198

[18] Martone-Rocha S, Piveli RP, Matté GR, Dória MC, Dropa M, Morita M, et al. Dynamics of *Aeromonas* species isolated from wastewater treatment system. J. Water Health 2010;8(4):703–711. doi: 10.2166/wh.2010.140 20705981

[19] Uljanovas D, Gölz G, Brückner V, Grineviciene A, Tamuleviciene E, Alter T, et al. Prevalence, antimicrobial susceptibility and virulence gene profiles of *Arcobacter* species isolated from human stool samples, foods of animal origin, ready-to-eat salad mixes and environmental water. Gut Pathog. 2021;13:76. doi: 10.1186/s13099-021-00472-y 34930425 PMC8686351

[20] Ferreira S, Queiroz JA, Oleastro M, Domingues FC. Insights in the pathogenesis and resistance of *Arcobacter*: A review. Crit. Rev. Microbiol. 2016;42(3):364–383. doi: 10.3109/1040841X.2014.954523 25806423

[21] Ghaju Shrestha R, Tanaka Y, Haramoto E. A review on the prevalence of *Arcobacter* in aquatic environments. Water 2022;14(8):1266 doi: 10.3390/w14081266

[22] Ghaju Shrestha R, Sherchan SP, Kitajima M, Tanaka Y, Gerba CP, Haramoto E. Reduction of *Arcobacter* at two conventional wastewater treatment plants in southern Arizona, USA. Pathogens 2019;8(4):175. doi: 10.3390/pathogens8040175 31581714 PMC6963474

[23] Matsen JM, Spindler JA, Blosser RO. Characterization of *Klebsiella* isolates from natural receiving waters and comparison with human isolates. Appl. Microbiol. 1974;28(4):672–678. doi: 10.1128/am.28.4.672-678.1974 4607526 PMC186795

[24] Struve C, Krogfelt KA. Pathogenic potential of environmental *Klebsiella pneumoniae* isolates. Environ. Microbiol. 2004;6(6):584–590. doi: 10.1111/j.1462-2920.2004.00590.x 15142246

[25] Knittel MD, Seidler RJ, Eby C, Cabe LM. Colonization of the botanical environment by *Klebsiella* isolates of pathogenic origin. Appl. Environ. Microbiol. 1977;34(5):557–563. doi: 10.1128/aem.34.5.557-563.1977 337900 PMC242700

[26] Wyres KL, Lam MMC, Holt KE Population genomics of *Klebsiella pneumoniae*. Nat. Rev. Microbiol. 2020;18:344–359. doi: 10.1038/s41579-019-0315-1 32055025

[27] Barbier E, Rodrigues C, Depret G, Passet V, Gal L, Piveteau P, et al. The ZKIR assay, a real-time PCR method for the detection of *Klebsiella pneumoniae* and closely related species in environmental samples. Appl. Environ. Microbiol. 2020;86(7):e02711–19. doi: 10.1128/AEM.02711-19 32005732 PMC7082575

[28] Shannon KE, Lee DY, Trevors JT, Beaudette LA. Application of real-time quantitative PCR for the detection of selected bacterial pathogens during municipal wastewater treatment. Sci. Total Environ. 2007;382(1):121–129. doi: 10.1016/j.scitotenv.2007.02.039 17462712

[29] Johansen MD, Herrmann J-L, Kremer L. Non-tuberculous mycobacteria and the rise of *Mycobacterium abscessus*. Nat. Rev. Microbiol. 2020;18:392–407. doi: 10.1038/s41579-020-0331-1 32086501

[30] To K, Cao R, Yegiazaryan A, Owens J, Venketaraman V. General overview of nontuberculous Mycobacteria opportunistic pathogens: *Mycobacterium avium* and *Mycobacterium abscessus*. J. Clin. Med. 2020;9(8):2541. doi: 10.3390/jcm9082541 32781595 PMC7463534

[31] Alexander KJ, Furlong JL, Baron JL, Rihs JD, Stephenson D, Perry JD, et al. Evaluation of a new culture medium for isolation of nontuberculous mycobacteria from environmental water samples. PLoS One 2021;16(3):e0247166. doi: 10.1371/journal.pone.0247166 33657154 PMC7928522

[32] Radomski N, Lucas FS, Moilleron R, Cambau E, Haenn S, Moulin L. Development of a real-time qPCR method for detection and enumeration of *Mycobacterium* spp. in surface water. Appl. Environ. Microbiol. 2010;76(21):7348–7351. doi: 10.1128/AEM.00942-10 20851986 PMC2976254

[33] Radomski N, Betelli L, Moilleron R, Haenn S, Moulin L, Cambau E, et al. *Mycobacterium* behavior in wastewater treatment plant, a bacterial model distinct from *Escherichia coli* and *Enterococci*. Environ. Sci. Technol. 2011;45(12):5380–5386. doi: 10.1021/es104084c 21591688

[34] World Health Organization. WHO guidelines for the safe use of wastewater, excreta and greywater—Volume 4 Excreta and greywater use in agriculture. Geneva: WHO press; 2006. https://www.who.int/publications/i/item/9241546859

[35] Rock CM, Brassill N, Dery JL, Carr D, McLain JE, Bright KR, et al. Review of water quality criteria for water reuse and risk-based implications for irrigated produce under the FDA Food Safety Modernization Act, produce safety rule. Environ Res. 2019;172:616–629. doi: 10.1016/j.envres.2018.12.050 30878733

[36] Schellenberg T, Subramanian V, Ganeshan G, Tompkins D, Pradeep R. Wastewater discharge standards in the evolving context of urban sustainability–The case of India. Front. Environ. Sci. 2020;8:30. doi: 10.3389/fenvs.2020.00030

[37] Water Environmental Partnership in Asia. Outlook on water environmental management in Asia 2021. Kanagawa: Institute for Global Environmental Strategies; 2021. https://wepa-db.net/wp-content/uploads/2023/02/WEPA_outlook2021_e.pdf

[38] Ahmed W, Huygens F, Goonetilleke A, Gardner T. Real-time PCR detection of pathogenic microorganisms in roof-harvested rainwater in Southeast Queensland, Australia. Appl. Environ. Microbiol. 2008;74(17):5490–5496. doi: 10.1128/AEM.00331-08 18621865 PMC2546628

[39] Wéry N, Lhoutellier C, Ducray F, Delgenès J-P, Godon J-J. Behaviour of pathogenic and indicator bacteria during urban wastewater treatment and sludge composting, as revealed by quantitative PCR. Water Res. 2008;42(1–2):53–62. doi: 10.1016/j.watres.2007.06.048 17659319

[40] Tomioka N, Yoochatchaval W, Takemura Y, Matsuura N, Danshita T, Srisang P, et al. Detection of potentially pathogenic *Arcobacter* spp. in Bangkok canals and the Chao Phraya River. J. Water Health 2021;19(4):657–670. doi: 10.2166/wh.2021.239 34371501

[41] Rodríguez S, Araujo R. Effect of environmental parameters on the inactivation of the waterborne pathogen *Campylobacter* in a Mediterranean river. J. Water Health 2012;10(1):100–107. doi: 10.2166/wh.2011.044 22361705

[42] Endo S, Koga S. Johkasou–Wastewater Management in a Local Municipality in Japan. Asian Development Bank Institute (ADBI) Development Case Study No. 2021–4 (October). Tokyo; 2021. https://www.adb.org/publications/johkasou-wastewater-management-japan

[43] Talang RPN, Sirivithayapakorn S, Polruang S. Environmental impacts and cost-effectiveness of Thailand’s centralized municipal wastewater treatment plants with different nutrient removal processes. J. Clean. Prod. 2020;256:120433. doi: 10.1016/j.jclepro.2020.120433

[44] Takemura Y, Yoochatchaval W, Danshita T, Miyaoka Y, Aoki M, Tran PT, et al. A pilot-scale study of a down-flow hanging sponge reactor as a post-treatment for domestic wastewater treatment system at short hydraulic retention times. J. Water Process Eng. 2022;50:103313. doi: 10.1016/j.jwpe.2022.103313

[45] Ludwig W, Strunk O, Westram R, Richter L, Meier H, Yadhukumar, et al. ARB: a software environment for sequence data. Nucleic Acids Res. 2004;32(4):1363–1371. doi: 10.1093/nar/gkh293 14985472 PMC390282

[46] Yilmaz P, Parfrey LW, Yarza P, Gerken J, Pruesse E, Quast C, et al. The SILVA and "All-species Living Tree Project (LTP)" taxonomic frameworks. Nucleic Acids Res. 2014;42(D1):D643–D648. doi: 10.1093/nar/gkt1209 24293649 PMC3965112

[47] Klindworth A, Pruesse E, Schweer T, Peplies J, Quast C, Horn M, et al. Evaluation of general 16S ribosomal RNA gene PCR primers for classical and next-generation sequencing-based diversity studies. Nucleic Acids Res. 2013;41(1):e1. doi: 10.1093/nar/gks808 22933715 PMC3592464

[48] Quast C, Pruesse E, Yilmaz P, Gerken J, Schweer T, Yarza P, et al. The SILVA ribosomal RNA gene database project: improved data processing and web-based tools. Nucleic Acids Res. 2013;41(D1):D590–D596. doi: 10.1093/nar/gks1219 23193283 PMC3531112

[49] Caporaso JG, Lauber CL, Walters WA, Berg-Lyons D, Lozupone CA, Turnbaugh PJ, et al. Global patterns of 16S rRNA diversity at a depth of millions of sequences per sample. Proc. Natl. Acad. Sci. USA 2011;108(supplement_1):4516–4522. doi: 10.1073/pnas.1000080107 20534432 PMC3063599

[50] Aoki M, Okubo K, Kusuoka R, Watari T, Syutsubo K, Yamaguchi T. Hexavalent chromium removal and prokaryotic community analysis in glass column reactor packed with aspen wood as solid organic substrate. Appl. Biochem. Biotechnol. 2022;194:1425–1441. doi: 10.1007/s12010-021-03738-y 34739702

[51] Martin M. Cutadapt removes adapter sequences from high-throughput sequencing reads. EMBnet J 2011;17(1):10–12. doi: 10.14806/ej.17.1.200

[52] Schloss PD, Westcott SL, Ryabin T, Hall JR, Hartmann M, Hollister EB, et al. Introducing mothur: open-source, platform-independent, community-supported software for describing and comparing microbial communities. Appl. Environ. Microbiol. 2009;75(23):7537–7541. doi: 10.1128/AEM.01541-09 19801464 PMC2786419

[53] Kozich JJ, Westcott SL, Baxter NT, Highlander SK, Schloss PD. Development of a dual-index sequencing strategy and curation pipeline for analyzing amplicon sequence data on the MiSeq Illumina sequencing platform. Appl. Environ. Microbiol. 2013;79(17):5112–5120. doi: 10.1128/AEM.01043-13 23793624 PMC3753973

[54] Westcott SL, Schloss PD. OptiClust, an improved method for assigning amplicon-based sequence data to operational taxonomic units. mSphere 2017;2(2):e00073–17. doi: 10.1128/mSphereDirect.00073-17 28289728 PMC5343174

[55] Yoon S-H, Ha S-M, Kwon S, Lim J, Kim Y, Seo H, et al. Introducing EzBioCloud: a taxonomically united database of 16S rRNA gene sequences and whole-genome assemblies. Int. J. Syst. Evol. Microbiol. 2017;67(5):1613–1617. doi: 10.1099/ijsem.0.001755 28005526 PMC5563544

[56] Mise K, Iwasaki W. Environmental atlas of prokaryotes enables powerful and intuitive habitat-based analysis of community structures. iScience 2020;23(10):101624. doi: 10.1016/j.isci.2020.101624 33117966 PMC7581931

[57] Japanese Standards Association. JIS K 0102:2016 Testing methods for industrial wastewater. Tokyo: The Association; 2016.

[58] Danshita T, Yoochatchaval W, Takemura Y, Miyaoka Y, Kada M, Tepjun W, et al. Performance evaluation of a down-flow hanging sponge (DHS) reactor as a decentralized domestic wastewater treatment system in tropical regions. J. Environ. Sci. Health A Tox. Hazard. Subst. Environ. Eng. 2020;55(7):847–857. doi: 10.1080/10934529.2020.1748472 32253973

[59] Kodaka H, Mizuochi S, Teramura H, Nirazuka T, Goins D, Odumeru J, et al. Comparison of the Compact Dry EC with the most probable number method (AOAC official method 966.24) for enumeration of *Escherichia coli* and coliform bacteria in raw meats: Performance-Tested Method^SM^ 110402. J. AOAC Int. 2006;89(1):100–114. doi: 10.1093/jaoac/89.1.100 16512235

[60] Revelle W. psych: Procedures for Psychological, Psychometric, and Personality Research. Version 2.2.9 [software]. Northwestern University. 2022 Sep 12. https://cran.r-project.org/web/packages/psych/

[61] Wei T, Simko V. corrplot: Visualization of a Correlation Matrix. Version 0.9.2 [software]. 2021 Nov 18. https://cran.r-project.org/web/packages/corrplot/

[62] R Core Team. R: A language and environment for statistical computing. Version 4.1.0 [software]. R Foundation for Statistical Computing. 2021 May 18. https://www.r-project.org/

[63] Pérez-Cataluña A, Salas-Massó N, Diéguez AL, Balboa S, Lema A, Romalde JL, et al. Revisiting the taxonomy of the genus *Arcobacter*: Getting order from the chaos. Front. Microbiol. 2018;9:2077. doi: 10.3389/fmicb.2018.02077 30233547 PMC6131481

[64] On SLW, Miller WG, Biggs PJ, Cornelius AJ, Vandamme P. *Aliarcobacter*, *Halarcobacter*, *Malaciobacter*, *Pseudarcobacter* and *Poseidonibacter* are later synonyms of *Arcobacter*: transfer of *Poseidonibacter parvus*, *Poseidonibacter antarcticus*, ’*Halarcobacter arenosus*’, and ’*Aliarcobacter vitoriensis*’ to *Arcobacter* as *Arcobacter parvus* comb. nov., *Arcobacter antarcticus* comb. nov., *Arcobacter arenosus* comb. nov. and *Arcobacter vitoriensis* comb. nov. Int. J. Syst. Evol. Microbiol. 2021;71(11):005133. doi: 10.1099/ijsem.0.005133 34825881

[65] McLellan SL, Roguet A. The unexpected habitat in sewer pipes for the propagation of microbial communities and their imprint on urban waters. Curr. Opin. Biotechnol. 2019;57:34–41. doi: 10.1016/j.copbio.2018.12.010 30682717 PMC7018504

[66] Greay TL, Gofton AW, Zahedi A, Paparini A, Linge KL, Joll CA, et al. Evaluation of 16S next-generation sequencing of hypervariable region 4 in wastewater samples: An unsuitable approach for bacterial enteric pathogen identification. Sci. Total Environ. 670:1111–1124. doi: 10.1016/j.scitotenv.2019.03.278 31018427

[67] Chen Y-J, Leung PM, Wood JL, Bay SK, Hugenholtz P, Kessler AJ, et al. Metabolic flexibility allows bacterial habitat generalists to become dominant in a frequently disturbed ecosystem. ISME J. 2021;15:2986–3004. doi: 10.1038/s41396-021-00988-w 33941890 PMC8443593

[68] Japanese Society for Bacteriology [Internet]. Tokyo: Biosafety levels of pathogenic bacteria (in Japanese). [cited 2023 Feb 3]. https://jsbac.org/archive/img/bsl_level.pdf.

[69] Levican A, Alkeskas A, Günter C, Forsythe SJ, Figueras MJ. Adherence to and invasion of human intestinal cells by *Arcobacter* species and their virulence genotypes. Appl. Environ. Microbiol. 2013;79(16):4951–4957. doi: 10.1128/AEM.01073-13 23770897 PMC3754706

[70] Wang G, Clark CG, Liu C, Pucknell C, Munro CK, Kruk TMAC, et al. Detection and characterization of the hemolysin genes in *Aeromonas hydrophila* and *Aeromonas sobria* by multiplex PCR. J. Clin. Microbiol. 2003;41(3):1048–1054. doi: 10.1128/JCM.41.3.1048-1054.2003 12624028 PMC150266

[71] Yu C-P, Farrell SK, Robinson B, Chu K-H. Development and application of real-time PCR assays for quantifying total and aerolysin gene-containing *Aeromonas* in source, intermediate, and finished drinking water. Environ. Sci. Technol. 2008;42(4):1191–1200. doi: 10.1021/es071341g 18351092

[72] Robertson BK, Harden C, Selvaraju SB, Pradhan S, Yadav JS. Molecular detection, quantification, and toxigenicity profiling of *Aeromonas* spp. in source- and drinking-water. Open Microbiol. J. 2014;8:32–39. doi: 10.2174/1874285801408010032 24949108 PMC4062929

[73] Kanchanapiya P, Tantisattayakul T. Wastewater reclamation trends in Thailand. Water Sci. Technol. 2022;86(11):2878–2911. doi: 10.2166/wst.2022.375 36515195

[74] Garcia-Aljaro C, Momba M, Muniesa M. Pathogenic members of *Escherichia coli* & *Shigella* spp. Shigellosis. In: Rose JB, Jiménez-Cisneros B, editors. Water and Sanitation for the 21st Century: Health and Microbiological Aspects of Excreta and Wastewater Management (Global Water Pathogen Project). Michigan State University, UNESCO; 2017.

[75] Wang M, Zhu J, Mao X. Removal of pathogens in onsite wastewater treatment systems: a review of design considerations and influencing factors. Water 2021;13(9):1990. doi: 10.3390/w13091190

[76] Zhang T, Shao M-F, Ye L. 454 pyrosequencing reveals bacterial diversity of activated sludge from 14 sewage treatment plants. ISME J. 2012;6:1137–1147. doi: 10.1038/ismej.2011.188 22170428 PMC3358032

[77] Guo F, Zhang T, Li B, Wang Z, Ju F, Liang Y. Mycobacterial species and their contribution to cholesterol degradation in wastewater treatment plants. Sci. Rep. 2019;9:836. doi: 10.1038/s41598-018-37332-w 30696864 PMC6351609

[78] Solaiman S, Allard SM, Callahan MT, Jiang C, Handy E, East C, et al. Longitudinal assessment of the dynamics of *Escherichia coli*, total coliforms, *Enterococcus* spp., and *Aeromonas* spp. in alternative irrigation water sources: a CONSERVE Study. Appl. Environ. Microbiol. 2020;86(20):e00342–20. doi: 10.1128/AEM.00342-20 32769196 PMC7531960

[79] Pavlik I, Ulmann V, Hubelova D, Weston RT. Nontuberculous mycobacteria as sapronoses: A review. Microorganisms 2022;10(7):1345. doi: 10.3390/microorganisms10071345 35889064 PMC9315685

[80] Berney M, Greening C, Conrad R, Jacobs WR Jr., Cook GM. An obligately aerobic soil bacterium activates fermentative hydrogen production to survive reductive stress during hypoxia. Proc. Natl. Acad. Sci. USA 2014;111(31):11479–11484. doi: 10.1073/pnas.1407034111 25049411 PMC4128101

[81] Zeng D, Chen Z, Jiang Y, Xue F, Li B. Advances and challenges in viability detection of foodborne pathogens. Front. Microbiol. 2016;7:1833. doi: 10.3389/fmicb.2016.01833 27920757 PMC5118415

[82] Marutescu LG, Popa M, Gheorghe-Barbu I, Barbu IC, Rodríguez-Molina D, Berglund F, et al. Wastewater treatment plants, an “escape gate” for ESCAPE pathogens. Front. Microbiol. 2023;14:1193907. doi: 10.3389/fmicb.2023.1193907 37293232 PMC10244645

[83] Zerva I, Remmas N, Kagalou I, Melidis P, Ariantsi M, Sylaios G, et al. Effect of chlorination on microbiological quality of effluent of a full-scale wastewater treatment plant. Life 2021;11(1):68. doi: 10.3390/life11010068 33477775 PMC7832327

