# Supplementary material for: Quantitative detection and reduction of potentially pathogenic bacterial groups of Aeromonas, Arcobacter, Klebsiella pneumoniae species complex, and Mycobacterium in wastewater treatment facilities
Source: PLoS One. 2023 Sep 28;18(9):e0291742. doi: 10.1371/journal.pone.0291742 (PMC10538766; doi:10.1371/journal.pone.0291742)
Supplement: S1 Text — (PDF) [file pone.0291742.s002.pdf]

S1 Text. Detailed methods of quantitative real-time polymerase chain reaction (qPCR) assays.

qPCR assays were performed on the LightCycler 96 system using FastStart Essential DNA Green Master (for *Aeromonas*, *Arcobacter*, and *Klebsiella pnuemoniae* species complex [KpSC] quantification) or FastStart Essential DNA Probes Master (for *Mycobacterium* quantification) (Roche Diagnostics GmbH, Mannheim, Germany). Reaction mixtures (20.0 μL in total) containing 1.0 or 2.0 μL of extracted DNA solution, 0.20–0.40 μM primers, 0.10 μM probe (used for *Mycobacterium* quantification only), and 1× FastStart Essential DNA Green Master or 1× FastStart Essential DNA Probes Master were prepared. The qPCR conditions (Table 2), including the oligonucleotide primer/probe concentrations and annealing temperatures, were determined empirically for each qPCR assay. In the qPCR assays using FastStart Essential DNA Green Master, post-amplification melting curve analysis (65–97 °C) was performed to confirm the presence of a single dominant melting curve peak derived from the target gene/sequence. We also confirmed that the qPCR assays did not produce unspecific amplification using 5 ng of mock DNA (DNA-Mock-001, Lot Number BK1912D01) obtained from the Biological Resource Center (National Institute of Technology and Evaluation (NBRC), Chiba, Japan). The mock DNA consisted of equal copy numbers of genomic DNA derived from 15 NBRC bacterial strains that are not taxonomically assigned to either *Aeromonas*, *Arcobacter*, KpSC, or *Mycobacterium*. A ten-fold dilution series of known concentrations of standard DNA fragments (S1 Table) and no-template controls were prepared for each run. The standard DNA fragment concentrations (ng μL^{-1}) were determined using the Qubit dsDNA HS Assay Kit and Qubit 4 Fluorometer (Thermo Fisher Scientific K.K., Tokyo, Japan) and converted to match the corresponding copy number of the target gene/sequence (copies μL^{-1}) using the Thermo Fisher Scientific DNA Copy

Number and Dilution Calculator. The coefficient of determination (R^2) was ≥0.99 for all standard curves. The copy numbers of the target gene/sequence in the analyzed samples were calculated based on the average concentrations of the duplicate PCR amplification results. The abundances of *Aeromonas*, *Arcobacter*, and *Mycobacterium* (cells mL^{-1}) in the analyzed samples were estimated by dividing the obtained 16S rRNA gene copy numbers by the median *rrn* operon copy number of the genome of the targeted bacterial groups. The median copy number was determined using *rrn*DB v.5.8 (*Aeromonas*: 10 copies, *Arcobacter*: 5 copies, *Mycobacterium*: 1 copy) [1]. To estimate KpSC abundance, one copy of the intergenic region sequence of zinc uptake regulator and hemolysin genes was converted to one KpSC cell. The obtained qPCR data were analyzed using the LightCycler 96 Application Software v.1.1 (Roche Diagnostics International Ltd).

The limit of detection (LoD) and limit of quantification (LoQ) of the qPCR assays were determined as described previously [2], with slight modifications. Briefly, real-time PCR amplification of 1, 10, 20, 30, 40, 50, and 60 copies $reaction^{-1}$ of the standard DNA fragments (8 replicates each) was performed. The LoD was defined as the amount at which at least two out of eight replicates were amplified, regardless of the standard deviation (SD) of the cycle quantification (Cq) and amplification efficiency. The LoQ was defined as the lowest amount of the standard DNA at which all eight replicates were amplified with an SD of Cq <0.8. The determined LoD and LoQ values (copies $reaction^{-1}$) were converted to sample volume-based LoD and LoQ values (cells mL^{-1}) by considering the maximum net sample volume for DNA extraction (8.0 mL), the elution volume of extracted DNA (50 μL), the maximum applied volume of extracted DNA solution for qPCR assays (2.0 μL), and the copy number of the target gene/sequence in the genome of the targeted bacterial groups, as mentioned above.

References for S1 Text

1. Stoddard SF, Smith BJ, Hein R, Roller BRK, Schmidt TM. *rrn*DB: improved tools for interpreting rRNA gene abundance in bacteria and archaea and a new foundation for future development. Nucleic Acids Res. 2015;43(D1):D593-D598. https://doi.org/10.1093/nar/gku1201
2. Price EP, Dale JL, Cook JM, Sarovich DS, Seymour ML, Ginther JL, et al. Development and validation of *Burkholderia pseudomallei*-specific real-time PCR assays for clinical, environmental or forensic detection applications. PLoS One 2012;7(5):e37723. https://doi.org/10.1371/journal.pone.0037723
