# Supplementary material for: Quantitative detection and reduction of potentially pathogenic bacterial groups of Aeromonas, Arcobacter, Klebsiella pneumoniae species complex, and Mycobacterium in wastewater treatment facilities
Source: PLoS One. 2023 Sep 28;18(9):e0291742. doi: 10.1371/journal.pone.0291742 (PMC10538766; doi:10.1371/journal.pone.0291742)
Supplement: S1 Table — (PDF) [file pone.0291742.s003.pdf]

S1 Table. Sequences of synthetic standard DNA fragments used for quantitative real-time polymerase chain reaction assays.

Target group	Synthetic standard DNA fragment sequence (5′ to 3′)	GenBank accession number
Aeromonas	AGAGTTTGATCATGGCTCAGATTGAACGCTGGCGGCA<br>GGCCTAACACATGCAAGTCGAGCGGCAGCGGGAAAGT<br>AGCTTGCTACTTTTGCCGGCGAGCGGCGGACGGGTGA<br>GTAATGCCTGGGGATCTGCCCAGTCGAGGGGGATAAC<br>TACTGGAAACGGTAGCTAATACCGCATACGCCCTACG<br>GGGGAAAGCAGGGGACCTTCGGGCCTTGCGCGATTGG<br>ATGAACCCAGGTGGGATTAGCTAGTTGGTGAGGTAAT<br>GGCTCACCAAGGCGACGATCCCTAGCTGGTCTGAGAG<br>GATGATCAGCCACACTGGAACTGAGACACGGTCCAGA<br>CTCCTACGGGAGGCAGCAGTGGGGAATATTGCACAAT<br>GGGGGAAACCCTGATGCAGCCATGCCGCGTGTGTGAA<br>GAAGGCCTTCGGGTTGTAAAGCACTTTCAGCGAGGAG<br>GAAAGGTTGGTAGCTAATAACTGCCAGCTGTGACGTT<br>ACTCGCAGAAGAAGCACCGGCTAACTCCGTGCCAGCA<br>GCCGCGGTAATACGGAGGGTGCAAGCGTTAATCGGAA<br>TTACTGGGCGTAAAGCGCACGCAGGCGGTTGGATAAG<br>TTAGATGTGAAAGCCCCGGGCTCAACCTGGGAATTGC<br>ATTTAAAACTGTCCAGCTAGAGTCTTGTAGAGGGGGGT<br>AGAATTCCAGGTGTAGCGGTGAAATGCGTAGAGATCT<br>GGAGGAATACCGGTGGCGAAGGCGGCCCCCTGGACAA<br>AGACTGACGCTCAGGTGCGAAAGCGTGGGGAGCAAAC<br>AGGATTAGATACCCTGGTAGTCCACGCCGTAAACGAT<br>GTCGATTTGGAGGCTGTGTCCTTGAGACGTGGCTTCCG<br>GAGCTAACGCGTTAAATCGACCGCCTGGGGAGTACGG<br>CCGCAAGGTTAAAACTCAAATGAATTGACGG	X74684
Arcobacter	AGAGTTTGATCCTGGCTCAGAGTGAACGCTGGCGGCG<br>TGCTTAACACATGCAAGTCGAACGAGAACGGATTATA<br>GCTTGCTATAATTGTCAGCTAAGTGGCGCACGGGTGAG<br>TAATGTATAGGTAATATGCCTCTTACTAAGGGATAACA<br>ATTGGAAACGATTGCTAATACCTTATATTCCTTTTTATC<br>AAAAGATAAAAAGGGAAAGATTTATTGGTAAGAGATT<br>AGCCTGTATTGTATCAGTTAGTTGGTGGGGTAATGGCC<br>TACCAAGACGATGACGCATAACTGGTTTGAGAGGATG<br>ATCAGTCACACTGGAACTGAGACACGGTCCAGACTCC<br>TACGGGAGGCAGCAGTGGGGAATATTGCACAATGGAC<br>GAAAGTCTGATGCAGCAACGCCGCGTGGAGGATGACA<br>CATTTCGGTGCGTAAACTCCTTTTATATAAGAAGATAA<br>TGACGGTATTATATGAATAAGCACCGGCTAACTCCGTG<br>CCAGCAGCCGCGGTAATACGGAGGGTGCAAGCGTTAC<br>TCGGAATCACTGGGCGTAAAGAGCGTGTAGGCGGATT<br>GATAAGTTTGAAGTGAAATCCTATAGCTTAACTATAGA<br>ACTGCTTTGAAAACTGTTAATCTAGAATGTGGGAGAG<br>GTAGATGGAATTTCTGGTGTAGGGGTAAAATCCGTAG<br>AGATCAGAAGGAATACCGATTGCGAAGGCGATCTACT<br>GGAACAATATTGACGCTGAGACGCGAAAGCGTGGGGA<br>GCAAACAGGATTAGATACCCTGGTAGTCCACGCCCTA<br>AACGATGTACACTAGTTGTTGTGAGGCTCGACCTTGCA<br>GTAATGCAGTTAACACATTAAGTGTACCGCCTGGGGA<br>GTACGGTCGCAAGATTAAAACTCAAAGGAATAGACGG	CP000361

S1 Table. (Continued)

Target group	Synthetic standard DNA fragment sequence (5′ to 3′)	GenBank accession number
Klebsiella pneumoniae species complex	GAGCATCCTCGGATTGCGTGAATTTGAATCTGTTACTT TATCACGTTCCTCAGAAAACACCGAGAGCCGGCAGGT GATACGGGTCATACTTCAGGTTGTCGGCACCGGCGCCC GTCCCGGGCGCTGGCCGCTTATTCTCGAGCAGACTACG CCTTAGCCGCTCGCTAAAACCACCATGTCCGATTTAAT CACAACACGCCCGACCCTGGGTATGCTATATCTGAAGT GTCTCATTTTCGGGAGAAAACG	JOOW00000000
Mycobacterium	AGAGTTTGATCCTGGCTCAGGACGAACGCTGGCGGCG TGCTTAACACATGCAAGTCGAACGGAAAGGCCTCTTC GGAGGTACTCGAGTGGCGAACGGGTGAGTAACACGTG GGCAATCTGCCCTGCACTTCGGGATAAGCCTGGGAAA CTGGGTCTAATACCGGATAGGACCTCAAGACGCATGT CTTCTGGTGGAAAGCTTTTGCGGTGTGGGATGGGCCCG CGGCCTATCAGCTTGTTGGTGGGGTGACGGCCTACCAA GGCGACGACGGGTAGCCGGCCTGAGAGGGTGTCCGGC CACACTGGGACTGAGATACGGCCCAGACTCCTACGGG AGGCAGCAGTGGGGAATATTGCACAATGGGCGCAAGC CTGATGCAGCGACGCCGCGTGGGGGATGACGGCCTTC GGGTTGTAAACCTCTTTCACCATCGACGAAGGTCCGGG TTTTCTCGGATTGACGGTAGGTGGAGAAGAAGCACCG GCCAACTACGTGCCAGCAGCCGCGGTAATACGTAGGG TGCGAGCGTTGTCCGGAATTACTGGGCGTAAAGAGCT CGTAGGTGGTTTGTCGCGTTGTTCGTGAAATCTCACGG CTTAACTGTGAGCGTGCGGGCGATACGGGCAGACTAG AGTACTGCAGGGGAGACTGGAATTCCTGGTGTAGCGG TGGAATGCGCAGATATCAGGAGGAACACCGGTGGCGA AGGCGGGTCTCTGGGCAGTAACTGACGCTGAGGAGCG AAAGCGTGGGGAGCGAACAGGATTAGATACCCTGGTA GTCCACGCCGTAAACGGTGGGTACTAGGTGTGGGTTTC CTTCCTTGGGATCCGTGCCGTAGCTAACGCATTAAGTA CCCCGCCTGGGGAGTACGGCCGCAAGGCTAAAACTCA AAGGAATTGACGG	GQ153272
