# Supplementary material for: Quantitative detection and reduction of potentially pathogenic bacterial groups of Aeromonas, Arcobacter, Klebsiella pneumoniae species complex, and Mycobacterium in wastewater treatment facilities
Source: PLoS One. 2023 Sep 28;18(9):e0291742. doi: 10.1371/journal.pone.0291742 (PMC10538766; doi:10.1371/journal.pone.0291742)
Supplement: S2 Table — (PDF) [file pone.0291742.s004.pdf]

S2 Table. ***In silico*** **coverage and specificity of primer sets targeting the 16S rRNA gene of the genus** ***Aeromonas*****.**

Primer set	Amplicon size (bp) [a]	Number of total eligible sequences	Number of eligible *Aeromonas* sequences	0 mismatch			1 mismatch			Reference
				Number of hits in *Aeromonas*	Coverage (%) [b]	Number of outgroup hits	Number of hits in *Aeromonas*	Coverage (%) [b]	Number of outgroup hits	
Aero581F/ Aero848R	268	458,414	1,030	765	74.3	7	830	80.6	8	This study
A16SF/ A16SR	356	438,123	983	658	66.9	13	706	71.8	17	[1]
Aer66f/ Aer613r	548	406,995	933	644	69.0	12	702	75.2	13	[2]

[a] Amplicon size of the 16S rRNA gene of *Aeromonas veronii* ATCC 35624 (GenBank accession number: X74684).

[b] Coverage refers to the percentage of matches for *Aeromonas* sequences.

References for S2 Table

1. Wang G, Clark CG, Liu C, Pucknell C, Munro CK, Kruk TMAC, et al. Detection and characterization of the hemolysin genes in *Aeromonas hydrophila* and *Aeromonas sobria* by multiplex PCR. J. Clin. Microbiol. 2003;41(3):1048-1054. https://doi.org/10.1128/JCM.41.3.1048-1054.2003
2. Yu C-P, Farrell SK, Robinson B, Chu K-H. Development and application of real-time PCR assays for quantifying total and aerolysin gene-containing *Aeromonas* in source, intermediate, and finished drinking water. Environ. Sci. Technol. 2008;42(4):1191-1200. https://doi.org/10.1021/es071341g
