# Supplementary material for: Quantitative detection and reduction of potentially pathogenic bacterial groups of Aeromonas, Arcobacter, Klebsiella pneumoniae species complex, and Mycobacterium in wastewater treatment facilities
Source: PLoS One. 2023 Sep 28;18(9):e0291742. doi: 10.1371/journal.pone.0291742 (PMC10538766; doi:10.1371/journal.pone.0291742)
Supplement: S3 Table — (PDF) [file pone.0291742.s005.pdf]

S3 Table. Overview of the water quality parameters and removal rates of COD_{Cr}, BOD, TN, SS, and VSS in the wastewater treatment facilities analyzed in this study (average ± standard deviation).

Facility	A		B		C	
	Wastewater influent	Treated effluent	Wastewater influent	Treated effluent	Wastewater influent	Treated effluent
Water quality parameter (unit)						
COD_{Cr} (mg L^{-1})	76 ± 13	47 ± 18	170 ± 90	18 ± 7	230 ± 50	15 ± 12
BOD (mg L^{-1})	74 ± 23	28 ± 8	83 ± 57	3.0 ± 0.7	140 ± 30	7 ± 13
TN (mg L^{-1})	28 ± 11	25 ± 9	36 ± 17	24 ± 6	42 ± 8	10 ± 7
NH_4^+-N (mg L^{-1})	14 ± 9	22 ± 10	24 ± 12	2.7 ± 2.3	30 ± 7	3 ± 6
NO_2^--N (mg L^{-1})	< 0.05	2.3 ± 2.4	0.3 ± 0.5	< 0.05	0.06 ± 0.08	0.15 ± 0.21
NO_3^--N (mg L^{-1})	0.5 ± 0.8	0.2 ± 0.2	3.0 ± 3.8	23 ± 5	0.3 ± 0.1	5.3 ± 1.4
SS (mg L^{-1})	110 ± 80	17 ± 8	80 ± 80	9 ± 4	110 ± 20	2 ± 2 [a]
VSS (mg L^{-1})	90 ± 70	14 ± 7	70 ± 80	8 ± 4	100 ± 20	1 ± 1 [a]
pH (-)	6.46 ± 0.23	7.02 ± 0.49	7.18 ± 0.23	5.80 ± 1.17	6.86 ± 0.24	6.78 ± 0.50
ORP (mV)	−121.6 ± 89.2	217.6 ± 144.4	−82.2 ± 76.4	28.5 ± 63.4	−78.5 ± 56.1	195.3 ± 91.8
DO (mg L^{-1})	1.59 ± 1.00	4.54 ± 1.60	1.46 ± 1.64	3.23 ± 1.33	3.13 ± 1.48	4.30 ± 2.18
Water temperature (°C)	27.6 ± 4.6	24.0 ± 4.7	22.2 ± 6.2	22.7 ± 5.2	21.2 ± 6.7	22.1 ± 7.1
Removal rate (%)						
COD_{Cr}	38 ± 22		85 ± 16		93 ± 7	
BOD	60 ± 13		95 ± 3		94 ± 11	
TN	11 ± 13		23 ± 28		77 ± 10	
SS	75 ± 21		85 ± 6		98 ± 2	
VSS	73 ± 23		84 ± 7		98 ± 2	

S3 Table. (Continued)

Facility	D		E		F	
	Wastewater influent	Treated effluent	Wastewater influent	Treated effluent	Wastewater influent	Treated effluent
Water quality parameter (unit)						
COD_{Cr} (mg L^{-1})	200 ± 80	48 ± 13	250 ± 90	40 ± 17	330 ± 400	12 ± 2
BOD (mg L^{-1})	130 ± 50	13 ± 4	130 ± 20	15 ± 14	160 ± 190	3.5 ± 1.4
TN (mg L^{-1})	34 ± 4	24 ± 3	34 ± 6	22 ± 8	23 ± 9	10 ± 4
NH_4^+-N (mg L^{-1})	24 ± 0	4.5 ± 3.4	21 ± 6	11 ± 11	10 ± 3	1.6 ± 1.6
NO_2^--N (mg L^{-1})	< 0.05	0.32 ± 0.08	< 0.05	0.10 ± 0.06	< 0.05	0.07 ± 0.10
NO_3^--N (mg L^{-1})	0.3 ± 0.1	17 ± 3	0.3 ± 0.1	9.9 ± 9.6	0.3 ± 0.3	8 ± 6
SS (mg L^{-1})	100 ± 40	14 ± 5	130 ± 30	19 ± 12	160 ± 250	6 ± 7
VSS (mg L^{-1})	90 ± 40	12 ± 4	130 ± 40	16 ± 9	140 ± 240	5 ± 6
pH (-)	7.18 ± 0.54	6.29 ± 0.44	6.97 ± 0.63	5.85 ± 1.68	6.49 ± 0.30	6.12 ± 0.19
ORP (mV)	66.2 ± 36.3	184.4 ± 102.9	110.4 ± 71.1	218.1 ± 86.0	−7.7 ± 63.7	140.2 ± 47.4
DO (mg L^{-1})	3.78 ± 1.72	1.90 ± 0.94	3.74 ± 1.11	1.95 ± 0.56	2.27 ± 0.56	1.77 ± 0.30
Water temperature (°C)	21.0 ± 4.8	22.0 ± 5.3	22.9 ± 4.9	24.8 ± 5.2	21.0 ± 5.0	21.6 ± 5.4
Removal rate (%)						
COD_{Cr}	75 ± 4		82 ± 11		90 ± 10	
BOD	90 ± 3		89 ± 11		95 ± 6	
TN	31 ± 5		30 ± 32		53 ± 17	
SS	84 ± 9		84 ± 11		86 ± 17	
VSS	84 ± 8		86 ± 9		88 ± 16	

S3 Table. (Continued)

Facility	G		H		I	
	Wastewater influent	Treated effluent	Wastewater influent	Treated effluent	Wastewater influent	Treated effluent
Water quality parameter (unit)						
COD_{Cr} (mg L^{-1})	69 ± 22	23 ± 6	100 ± 50	16 ± 3	90 ± 40	21 ± 2
BOD (mg L^{-1})	56 ± 30 [b]	5.1 ± 1.9 [b]	64 ± 10 [b]	5.0 ± 0.9 [b]	54 ± 11[b]	6.7 ± 0.8 [b]
TN (mg L^{-1})	30 ± 10	12 ± 10	10 ± 7	5.3 ± 1.1	14 ± 1	10 ± 8
NH_4^+-N (mg L^{-1})	10 ± 7	3.2 ± 3.0	6.5 ± 1.6	0.8 ± 0.8	10 ± 2	2.5 ± 2.0
NO_2^--N (mg L^{-1})	0.016 ± 0.009	0.09 ± 0.16	0.012 ± 0.010	0.035 ± 0.039	0.002 ± 0.002	0.29 ± 0.25
NO_3^--N (mg L^{-1})	0.5 ± 0.4	3.3 ± 2.1	< 0.1	3.3 ± 2.1	0.2 ± 0.1	4.6 ± 0.8
SS (mg L^{-1})	23 ± 6	12 ± 4	38 ± 11	13 ± 5	15 ± 8	6 ± 2
VSS (mg L^{-1})	15 ± 5	8 ± 2	13 ± 4	5 ± 1	10 ± 4	3 ± 2
pH (-)	6.8 ± 0.5	6.7 ± 0.4	6.7 ± 0.3	6.8 ± 0.3	6.5 ± 0.3	6.6 ± 0.3
ORP (mV)	−114 ± 65	−16 ± 45	−127 ± 77	−26 ± 34	−149 ± 124	−19 ± 29
DO (mg L^{-1})	2.64 ± 1.89 [c]	4.28 ± 0.52 [c]	3.62 ± 0.68	4.21 ± 0.87	2.91 ± 1.78	5.02 ± 0.14
Water temperature (°C)	26.6 ± 1.4	26.9 ± 1.2	26.6 ± 2.0	29.9 ± 5.9	26.3 ± 1.6	26.9 ± 1.4
Removal rate (%)						
COD_{Cr}	65 ± 11		81 ± 10		73 ± 13	
BOD	89 ± 6		92 ± 2		87 ± 3	
TN	−9 ± 81		36 ± 27		30 ± 52	
SS	47 ± 21		67 ± 5		54 ± 15	
VSS	49 ± 15		61 ± 10		64 ± 18	

[a] Data for the treated effluent sample collected in November 2021 were missing.

[b] Allylthiourea, a nitrification inhibitor, was used in the measurement.

[c] Data for the influent wastewater and treated effluent samples collected in December 2021 were missing.

Abbreviations: COD_{Cr}, chemical oxygen demand determined by the potassium dichromate method; BOD, biochemical oxygen demand; TN, total nitrogen; NH_4^+-N, ammonium-nitrogen; NO_2^+-N, nitrite-nitrogen; NO_3^--N, nitrate-nitrogen; SS, suspended solids; VSS, volatile suspended solids; ORP, oxidation-reduction potential; and DO, dissolved oxygen.
